# Enhancing yogurt health benefits with moringa and black seed oil nanoemulsions to improve fatty acids and microbial viability

**DOI:** 10.1038/s41598-025-22091-2

**Published:** 2025-10-31

**Authors:** Hagar S. Abd-Rabou, Tarek Nour Soliman, Amira M. Galal Darwish, Sameh Awad, Marwa G. Allam

**Affiliations:** 1 Food Technology Department, Arid Lands Cultivation Research Institute, The City of Scientific Research and Technological Application (SRTA- City), Alexandria, Egypt; 2 Dairy Department, Food Industries and Nutrition Research Institute, National Research Centre, 12622 Dokki, Giza Egypt; 3 Food Industry Technology Program, Faculty of Industrial and Energy Technology, Borg Al Arab Technological University (BATU), Alexandria, Egypt; 4 Department of Dairy Science, Faculty of Agriculture, Alexandria University, El-Shatby, Alexandria, Egypt; 5 Food Science Department, Faculty of Agriculture, Saba Basha, Alexandria University, Alexandria, Egypt

**Keywords:** Antioxidant activity assessment, Fortified dairy products, Lactic acid bacteria viability, Nanoemulsion stabilization, Phenolic profiling of seed oils, Textural properties of yoghurt, Lipids, Quality of life

## Abstract

The increasing consumer demand for natural functional foods has prompted the development of fortified dairy products with improved nutritional and health benefits. This study designed and evaluated yoghurts fortified with cold-pressed moringa seed oil (MSO) and black seed oil (BSO) at 1.5 g /L, as well as their water-based nanoemulsions at 3 g Nanoemulsion powder/L, compared to a plain control. Phenolic profiles characterized by HPLC-MS revealed high levels of ellagic acid (23.5 mg/100 g), rutin (18.2 mg/100 g), and chlorogenic acid (21.4 mg/100 g) in MSO, and chlorogenic acid (19.8 mg/100 g), apigenin (16.7 mg/100 g), and naringenin (14.3 mg/100 g) in BSO. Nanoemulsions with 5% oil showed droplet sizes of 69.1 nm (MSO) and 38.1 nm (BSO) and zeta potentials above − 30 mV, confirming good colloidal stability over 7 days. Cytotoxicity assays indicated a safe dose up to 80 µg/mL. Yoghurt fortified with nanoemulsions exhibited a significant increase in total solids (+ 12%), unsaturated fatty acids (notably omega-3 increased by 25%), and antioxidant capacity (DPPH radical scavenging improved by 30%) while reducing acidity and syneresis relative to the control. Lactic acid bacteria viability remained unaffected. Sensory evaluation showed improved color, texture, and overall acceptance for yoghurts with 1.5 g nanoemulsion addition per liter. These findings demonstrate that incorporation of MSO and BSO nanoemulsions at this level effectively enhances yoghurt’s nutritional and functional properties without compromising microbial or sensory quality.

**Supplementary Information:**

The online version contains supplementary material available at 10.1038/s41598-025-22091-2.

## Introduction

Functional foods growing demand encourages the promotion of the beneficial food for human health by health professionals and nutritionists. Awareness of this is influencing an increase in the range of functional foods especially dairy products that contain essential fatty acids (EFAs), which are not synthesized in the human body, so they must be supplied with food. Deficiencies of EFAs in the diet can lead to the development of many diseases especially cardiovascular diseases and cancer. According to FAO/WHO appropriate ratio of n-3 and n-6 acids fatty acids should be supplied in the diet. Some vegetable oils show a nutritionally beneficial ratio of n-6 to n-3 fatty acids, which is also a source of polyphenols, tocopherols, and carotenoids, while milk and dairy products show a low content of n-3 fatty acids that can be enhanced by fortification^1,2^.

Oils fortifications are recommended as rich source of phytochemicals and unsaturated fatty acids that enrich the nutritional value. *Nigella sativa* which is often known as black cumin or black seed is an annual flowering plant. The seeds of black seed are interesting as they contain considerable amounts of phytochemicals with antioxidant qualities and health benefits. The seeds have a high concentration of fixed oil, which benefits both human health and nutrition owing to the presence of both major (essential fatty acids) and minor substances (phenolic compounds, tocopherols, and sterols). Tocols (tocopherols and tocotrienols) protect the oil from lipid oxidation and enhance its oxidative stability. Among the minor chemicals, thymoquinone is the main active component of the seeds and has several beneficial characteristics, including antioxidant and anti-inflammatory capabilities^3^. *Nigella sativa* oil is suggested in food applications as functional ingredient with high biological activities due to the presence of bioactive compounds^4^.

*oleifera* Lam. belongs to a single genus family Moringaceae which has 14 species. It is one of the most economically important species indigenous to dry tropical areas. Moringaceae family was utilized by the ancient Egyptians, Romans and Greeks. The seed oil contains all fatty acids contained in olive oil. In Egypt, the oil obtained from seeds of Moringa trees were used as edible oil sources in south cost of Red Sea region. *Moringa oleifera* seed oil is high in oleic acid and resembles in context of fatty acid composition with seed oils of other Moringa species. High-oleic oils are gaining importance, especially for replacing polyunsaturated vegetable oils and are reported to exhibit good oxidative stability during frying^5^.

Due to the hydrophobic nature of essential oils which makes them immiscible with cultured milk for yoghurt production, the use of edible nanoemulsions as delivery systems for lipophilic active substances has emerged. The small droplet size of nanoemulsion leads to less separation and aggregation impaired with high stability and reactivity of macromolecules. Oil-in-water nanoemulsion can easily be added to dairy products to improve its nutrition value. Nanoemulsions were added to several food system due to their functional properties in food processing^4,6^.

The fortification of dairy products, particularly yoghurt, with various functional oils has emerged as an important area of research aimed at enhancing nutritional value and providing health benefits. A significant number of studies have focused on diverse oils such as flaxseed oil^7^, fish oil^8^, chia seed oil^9^, and olive oil^10^. These investigations emphasize aspects like improving the fatty acid profile, increasing antioxidant capacity, and bolstering probiotic viability. For instance, the incorporation of fish oil nanoemulsion into yoghurt has been shown to affect its oxidative stability, which is crucial for ensuring that the benefits of omega-3 fatty acids are realized without compromising sensory attributes due to oxidation^8^.

The objective of this study was to develop and characterize fortified yoghurt products using cold-pressed moringa and black seed oils and their nanoemulsions, and to assess their influence on the physicochemical, microbiological, and functional properties of yoghurt.

## Materials and methods

### Materials

Commercial whey protein isolate (WPI) BiPro® was obtained from Davisco Foods International Inc. (Le Sueur, MN, USA) as a gift not general. It contains ≥ 97% protein on a dry basis, calculated using Kjeldahl *N*×6.38. The seed oil of *Moringa oleifera* was cordially obtained from the Egyptian Scientific Society of Moringa, National Research Center, Dokki, Cairo, Egypt and Black seed (*Nigella sativa*) was obtained from (Haraz) local market, Cairo, Egypt. Freeze-dried lactic culture YC-X11 (*Lactobacillus delbrueckii* subsp. *bulgaricus* and *Streptococcus thermophiles*) was obtained from Christian Hansen, Denmark. All phenolic standards, solvents and reagents were of HPLC grade (purity ≥ 98%; Sigma-Aldrich, Merck, Germany).

### Oil extraction

The Moringa (*Moringa oleifera*)*/* Black seed (*Nigella sativa*) oil extraction was performed applying the cold press technique using screw pressing without heating according to Tarasevičienė et al.,^3^, at Food industries and nutrition Research Division, National Research Centre (NRC), Dokki, Cairo, Egypt, according to local food safety standards and hygienic conditions. The collected oil filtered, packed in dark brown bottles, and stored at − 20 °C until use.

### Phenolic content via high performance liquid chromatography (HPLC)

Separation and quantitative determination of polyphenols content of extracted oil samples was carried out using HPLC apparatus model 1100 (Agilent Technologies, CA, USA) system column: Agilent Eclipse XDB C18 (150 × 4.6 μm; 5 μm), utilizing a UV-Visible Diode Array detector (DAD/UV) at a wavelength of 280 nm to separate and quantification of the phenolic compounds, according to Croci et al.,^11^. The used standards were: gallic acid, chlorogenic acid, catechin, methyl gallate, caffeic acid, syringic acid, pyrocatechol, rutin, ellagic acid, coumaric acid, vanillin, ferulic acid, naringenin, daidzein, quercetin, cinnamic acid, apigenin, kaempferol, and hesperetin.

### Preparation of nanoemulsion

Oil-in-water emulsions were prepared as described by Hamed and coauthors^8^, by homogenizing 5 and 10% w/w) lipid phase (moringa seed oil/ Black seed oil) with the aqueous phase containing 10% w/w) whey protein isolate as an emulsifier and completed to 100%.A coarse emulsion premix was formulated by blending the lipid and aqueous phases together using a high-speed homogenizer (Ingenieubüro CAT, M. Zipperer Gmbh, Germany) at 25000 rpm for 2 min at room temperature.

The coarse emulsion was further emulsified using a 160 W power, 20 kHz frequency, and 50% pulse (Sonic Vibra cell USA) for 15 min with 20 s time intervals. Increases in temperature during ultra-sonication are controlled by placing the sample container in a bigger beaker containing ice.

### Characterization of nanoemulsion

#### Particle size and zeta potential

The droplet diameter, zeta potential, and polydispersity index (PDI) which is used as a measure of the breadth of the molecular weight distribution, were measured using dynamic light scattering (Zetasizer, ZS, model 3600 Malvern Instruments, Malvern, UK) with Auto-measure software version 3.1 (Malvern Instruments). Prior to any measurements being taken, the samples were diluted 250 folds in twice-distilled water to avoiding multiple scattering effects^8^.

#### Creaming stability

After homogenization, 10 mL of nano-emulsions were straightaway transferred into a test tube with 0.1 sodium azide, to prevent microbial growth, sealed tightly, and stored for 7 days at room temperature (25 ± 2 °C)^12^. The stability of the creaming was measured through observing visually and subsequently calculated using the following equation:

1 $${\text{\% Creaming~index}}={\text{~}}\left( {\frac{{{\text{HL}}}}{{{\text{HE}}}}} \right) \times {\text{~}}100{\text{\% ~~}}$$

Where; HE = the overall height of emulsions, and HL = the total height of the layer.

#### Microstructure of nanoemulsion

Nanoemulsions samples were prepared for transmission electron microscopy (TEM). The samples were diluted (1:100 v/v) with deionized water. A drop of the diluted suspension was placed on the format-coated electron microscopy grid, left for 1 min, and then a drop of phosphotungstic acid solution (2% at pH 7.2) was added. The grid was air-dried and examined by TEM using a JEOL JEM-1400 plus TEM with an accelerating voltage of 100 kV at a magnification of 200,000 X^13^.

#### Cytotoxicity via hemolytic activity assay

The test was performed in 2 mL micro-tubes following the method described by Farias and coauthors^14^, with some modifications. Firstly, a twofold serial dilution of each extract was prepared with 0.9% NCl ranging from 1,000 to 1.9 μg/mL and reserved. Then, 100 μL of a 1% red blood cells (A, B, and O human blood types or rabbit blood) suspension was added to a new micro-tube containing 900 μL of each extract dilution, which was then taken to incubator at 37∘C for 1 h. After that, the tubes were centrifuged at 3,000 ×g for 5 min. The supernatant (200 μL) was placed in a 96-well plate and led to a microplate reader to measure the absorbance at 540 nm. The cell suspensions of each human blood type or those of rabbits (100 μL) were mixed with distilled water or 0.9% NCl (900 μL) to obtain the absorbance for 100 and 0% o cell lysis, respectively. The percentage of hemolysis was calculated as follows:

2 $$\% ~Hemolysis~=~\frac{{Abs~test}}{{Abs~pc~}} \times ~100~$$

Where; Abs test = Abs 540 nm of the 1% cell suspension treated with sample test, and Abs pc = Abs 540 nm of the 1% cell suspension treated with distilled water.

To calculate the relation between percentages of hemolysis and extract concentration, the hemolytic activity was expressed as the lowest seed extract concentration (μg/mL) capable of causing hemolysis ≥ 50%. All determinations were run in triplicate.

### Yoghurt preparation

Cow milk was standardized using skimmed milk powder (SMP) to SNF (15.5%), divided into five batches (1.0 L each). The first portion control plain yogurt without additives (T1), the second portion was fortified with 1.5 mL MSO/L (T2), the third portion was fortified with 3 g MSO-nanoemulsion, which contained 1.5 g MSO (T3), the fourth portion fortified with 15 mL BSO/L milk (T4), and the fifth fortified with 3 g BSO-nanoemulsion, which contained 1.5 g BSO (T5). The mixtures were stirred gently using magnetic stirrer to guarantee oil extracts/ nanoemulsion homogeneity. The fortified milk was then heat-treated at 85 ˚C for 10 min followed by cooling to 45 ˚C. The 5 portions were individually inoculated with (0.02 g/kg) freeze-dried lactic culture YC-X11 (*Lactobacillus delbrueckii* subsp. *bulgaricus* and *Streptococcus thermophiles*), poured into 50 mL plastic cups and incubated at 42 ± 1˚C until set coagulation, then cooled and stored at 4 °C^15^.

### Physicochemical properties of fortified yoghurt

Total solids (TS), protein, fat, pH and acidity of the yoghurt samples were determined according to AOAC^16^.

#### Water holding capacity (WHC) and syneresis

Twenty grams of yogurt samples (T1 control, T2, T3, T4 and T5) were placed on a filter paper resting on a top of a funnel. After 4 h of drainage at 7 o C, the quantity of whey collected in 10 mL graduated cylinder was used as an index of syneresis^17^. For syneresis weighed and stated as percent weight qualified to main weight of yogurt (20 g), while the water absorption capacity was determined by the below equation:

3 $$WHC~\left( {g~{H_2}O} \right)=~\left( {\frac{{W2~ - ~W1}}{{W0}}} \right) \times ~100~$$

Where; W0 is the weight of yogurt (g), W1 the weight of the tube plus yogurt (g). W2 weight of the tube plus the sediment (g).

#### Color analysis

Color analysis of yoghurt samples was conducted via Hunter colorimeter (Hunter Ultra Scan VIS). Values were expressed by Hunter *L*,* a*, and *b* values where, L* value of the lightness, as 0-100 representing dark to light, a* value of the degree of red and green color where higher positive indicating more red, and b* value of the degree of the yellow and blue colors, where higher value indicating more yellow^18^.

### Fatty acids profile via gas chromatography/mass spectrometry (GC-MS) analysis

GC-MS Analysis of fatty acids profile was applied to analyze the volatile compounds of the fortified nanoemulsions/ oils according to the method described by Bianchi et al.,^19^. The analysis was conducted to fresh fortified Yoghurt samples and the stored samples for 15 days. Compounds identification was carried out by comparing the retention times of those compounds by others obtained from NIST107, NIST21, and SZTERP libraries.

### Functional properties

#### Antioxidant potentials

The 2,2-diphenyl-1-picrylhydrazyl (DPPH) assay was performed as described by Gebremeskal et al.,^20^. Antioxidant activity was expressed as IC_50_ (mg/mL), where inhibition percent of DPPH radical is 50%.

#### Prevention of lipid oxidation

Peroxide value (PV) of the yogurt samples was determined according to official method AOCS (2009) (method Cd8-53)^21^ by titration a standard sodium thiosulfate (0.01 N) and was calculated as mL equivalent peroxides per kilogram sample (meqO_2_/Kg sample).

### Microbiology examination

Ten grams of each yogurt sample were dissolved in 90 mL of saline solution and a serial dilution was prepared. Then microbiological enumeration (count) was evaluated in each batch as follows: The total bacterial counts were plated on nutrient agar medium and incubated at 30 C for 48 h under aerobic condition (IDF, 1997). The total coliform count was determined using violet red bile agar medium. The plates were incubated at 37 °C for 18 h^22^ .

Total Lactic acid bacterial (LAB) counts were determined using MRS agar pH 5.5 (Biolife, Italy). The plates were incubated at 37 °C for 48 h under anaerobic condition. Yeasts and molds counts were determined using chloramphenicol rose bengal medium. The plates were incubated upside down at 22 °C for 5 days as described by Standard Methods for the Examination of Dairy Products^22^. The results were calculated directly as colony forming unit (CFU/g).

### Microstructure of yoghurt samples via scan electron microscope (SEM)

For preparation of Yoghurt samples, cubes (3 ± 0.5 mm) were cut from different areas of the yoghurt cup and fixed in 3% glutaraldehyde in 0.05 M phosphate buffer pH 7 for 2 h. at 48 °C. The fixed cubes were rinsed with 0.05 M phosphate buffer. The fixed cubes were dehydrated by consecutive soaking in 30, 50, 70 and 95% ethanol each for 20 min, and finally was rinsed successively twice by absolute ethanol (100%) at 48 °C and 58 °C. Cubes were immediately dried in the critical point drier (Samdri PVT-3B, Tousimis, Rockville, MD) for 5 h according to Vardhanabhuti et al.,^23^. The prepared yoghurt samples were analyzed using a scanning electron microscope (SEM- Joel Jsm 6360LA, Japan) after the surfaces were vacuum coated with gold^24^.

### Texture profile analysis (TPA)

TPA of yoghurt samples was assessed using a texture analyzer (TA1000, Lab Pro (FTC TMS-Pro), USA). Samples were tested in their cups using TA 17 probe (30 mm height and 25 mm diameter), after being allowed to stand at ambient temperature for at least 1 h. Two-bite penetration test was performed and operated at a crosshead speed of 1 mm/sec and a penetration distance of 10 mm. Firmness, consistency, cohesiveness and index of viscosity were evaluated as described by Bourne and coauthors & Szczesniak^25,26^ when fresh and after 15 days of cold storage.

### Sensory evaluation

Ten panelists (six men and four women, aged between 27 and 51 years) conducted a sensory evaluation of fresh and 15-day stored fortified yogurt samples versus plain yogurt at the Food Technology Dept., Arid Lands Cultivation Research Institute, SRTA-City, Alexandria, Egypt, as described by Darwish and coauthors^15^ with some modifications. The criteria for selection depended on their experience and background related to yogurt. The samples, stored at 4 °C, were allowed to rest at room temperature (25 °C) for 10 min before evaluation. The samples were evaluated using a 10-point Hedonic scale. This scale consisted of the test parameters of appearance, taste, consistency, odor, and overall acceptability, accompanied by a scale of ten categories as follows: 1 = dislike extremely; 2 = dislike much; 3 = dislike moderately; 4 = dislike slightly; 5 = neither dislike nor like; 6 = like slightly; 7 = like moderately; 8 = like much; 9 and 10 = like extremely.

### Statistical analysis

All data were expressed as mean values ± SD. Statistical analysis was performed IBM SPSS version 16.0 was used to examine the data given into the computer^27^. Statistical analysis was performed using one-way analysis of variance (ANOVA) followed by Duncan’s test. The obtained results were deemed significant at *P* < 0.05^28^.

## Results and discussion

### Phenolic content of extracted moringa and black seed oils (HPLC)

Table 1 revealed the contents of 19 polyphenols standard compounds in moringa and black seed oils namely; gallic acid, chlorogenic acid, catechin, methyl gallate, caffeic acid, syringic acid, pyro catechol, rutin, ellagic acid, coumaric acid, vanillin, ferulic acid, naringenin, daidzein, quercetin, cinnamic acid, apigenin, kaempferol, hesperetingallic acid. Those compounds were partially identified by the comparison of their retention times to those of authentic standards analyzed under identical conditions. The results indicate the rich content of the cold pressed black seed oil in phenolic compounds especially in; chlorogenic acid (9729.08 µg/mL), apigenin (4340.79 µg/mL), naringenin (4211.32 µg/mL), kaempferol acid (3986.72 µg/mL), ferulic acid (2469.89 µg/mL), caffeic acid (1031.30 µg/mL), rutin (1019.71 µg/mL), methyl gallate (1014.75 µg/mL), gallic acid (557.79 µg/mL), quercetin (556.20 µg/mL), catechin (467.38 µg/mL), ellagic acid (371.41 µg/mL), vanillin (228.72 µg/mL), and daidzein (157.06 µg/mL), in descending order. These results were in agreement with what previously reported by^29^. While in moringa oil the high phenolic compounds content was pronounced in; ellagic acid (779.14 µg/mL), rutin (589.05 µg/mL), chlorogenic acid (494.67 µg/mL), gallic acid (326.24 µg/mL), daidzein (226.27 µg/mL), cinnamic acid (219.49 µg/mL), naringenin (149.15 µg/mL), quercetin (134.64 µg/mL), syringic acid (133.57 µg/mL) and caffeic acid (112.17 µg/mL) in descending order. These results are in agreement with^30^. The obtained results emphasize the impact of cold press for producing high-quality moringa and black seed oils that was reflected in their rich active compounds content^31^. Additionally, the rich phenolic compounds content in both MSO and BSO can explain their antioxidant potentials illustrated in Figs. 4 and 5.

**Table 1:** Phenolic compound profile of cold-pressed Moringa seed oil and black seed oil extracts determined by HPLC.

Phenolic/ flavonoid	Moringa seed oil concentrations (µg/g)	Black seed oil concentrations (µg/g)
Gallic acid	326.24	557.79
Chlorogenic acid	494.67	9729.08
Catechin	32.24	467.38
Methyl gallate	69.83	1014.75
Caffeic acid	112.17	1031.30
Syringic acid	133.57	85.46
Pyro catechol	81.44	22.62
Rutin	589.05	1019.71
Ellagic acid	779.14	371.41
Coumaric acid	18.02	67.43
Vanillin	45.79	228.72
Ferulic acid	56.62	2469.89
Naringenin	149.15	4211.32
Daidzein	226.27	157.06
Quercetin	134.64	556.20
Cinnamic acid	219.49	93.05
Apigenin	12.17	4340.79
Kaempferol	ND	3986.72
Hesperetin	51.77	ND

ND: Not detected.

### Physical properties of nanoemulsions

The stability of nanoemulsions is influenced by several crucial factors: droplet size, zeta potential, and polydispersity index (PDI). Before determining the nanoemulsion properties, it is necessary to ensure the successful preparation of the nanoemulsion. The amphiphilic property of whey protein isolate, which includes the presence of both hydrophilic and hydrophobic groups within a single molecule, makes it a valuable food emulsifier^8,32^. The hydrophobic group of whey protein can bind to the oil phase, establishing molecular interactions between the protein molecules and the surfaces of the oil droplets. In contrast, the hydrophilic component of the system extends into the continuous aqueous phase, thereby minimizing droplet agglomeration or aggregation through the combined effects of steric and electrostatic repulsion^8^. The emulsion is prepared through a two-step homogenization procedure involving a high-speed and ultrasonic homogenizer. The ultrasonic homogenizer utilizes mechanical vibration to produce tiny cavitation and small droplets. When whey protein isolate (WPI) is employed as a surfactant, its molecules form a layer that promotes the equilibrium of interfacial tension, enhances the stability and protection of the internal phase (oil)^33^.

Table 2 presents the droplet size measurements of nanoemulsions containing concentrations of 5% and 10% (w/w) moringa and black seed oil. 5% BSO in the aqueous phase showed small droplet size of 38.09 ± 6.28 with PDI of 0.227 ± 0.017 which increased in the 5% MSO to 69.06 ± 8.56 nm, with a PDI of 0.302 ± 0.05. The same trend was observed in 10% MSO and BSO. These results are consistent with the results reported by^34^, who indicated that the particle size of the WPI nanoemulsion was 77.74 nm, with a PDI of 0.217. Hamed and coauthors^8^ observed that the size of the droplets in the nanoemulsion exhibited an increase as the oil concentration was raised^34^. stated that globular whey proteins can form robust, cohesive, and viscoelastic films around droplets. This property enables the formation of nanoemulsion droplets at a microscopic scale, which is highly resistant to droplet recoalescence and aggregation.

**Table 2:** Physicochemical properties of MSO or BSO nanoemulsions.

Nanoemulsion	Size (nm)	PDI	Zeta Particles (mV)	Creaming index
5% MSO	69.06^c^ ± 8.56	0.302^b^ ± 0.001	− 35.80^a^ ± 7.93	0.0 ± 0.0
10% MSO	135.90^b^ ± 19.70	0.385^a^ ± 0.003	− 50.50^b^ ± 7.50	0.0 ± 0.0
5% BSO	38.09^d^ ± 6.28	0.227^d^ ± 0.001	− 43.20^ab^ ± 9.17	0.0 ± 0.0
BSO	118.30^a^ ± 22.28	0.235^c^ ± 0.002	− 59.80^c^ ± 6.89	0.0 ± 0.0

Data are represented as means ± SD.

MSO, Moringa seed oil; BSO, Black seed oil.

The stability of nanoemulsions depends on the droplet size distribution and distribution (PDI)^36^. Based on the obtained results, the PDI for all treatments was within excellent limits for good emulsion stability. The PDI exhibited a range of values between 0.227 and 0.385. The electrokinetic potential in colloidal systems, commonly called zeta potential, is a term used to describe this phenomenon^34^. The above study agreed with^37^, examines the quantification of the disparity between the dispersion medium of the continuous liquid phase and the stationary layer associated with the dispersed particle in colloidal systems. The zeta potential value is associated with the stability of colloidal dispersions (emulsions), as it reflects the extent of repulsion between two phases and particles that possess similar charges. Table 2 presents the impact of varying concentrations of BSO and MSO on the zeta potential of the prepared nanoemulsions. It can be observed that the surface charge of 10% BSO exhibted a significantly high zeta potential value of -59.80 ± 6.89 mV, followed by a zeta potential value of -50.50 ± 7.50 mV for the 10% MSO. Addiionally, it was observed that both 5% MSO and BSO exhibited a decrease in surface charge. Furthermore, BSO displayed a more significant negative charge compared to MSO^37^. stated that a zeta potential value of 30 mV, regardless of its positive or negative, indicates good colloidal stability. Adding 10% WPI emulsifies has been observed to enhance the rapid and comprehensive coverage of recently formed droplet surfaces^36^. Furthermore, the amplification of electrostatic repulsion between the droplets would prevent their aggregation due to the significant surface charge exhibited by the droplets at a concentration of 10% BSO. Further invetigations of the nanoemulsion stability are also assessed through identifying destabilization mechanisms of creaming stability capabilities.

### Creaming stability

**Fig. 1:**
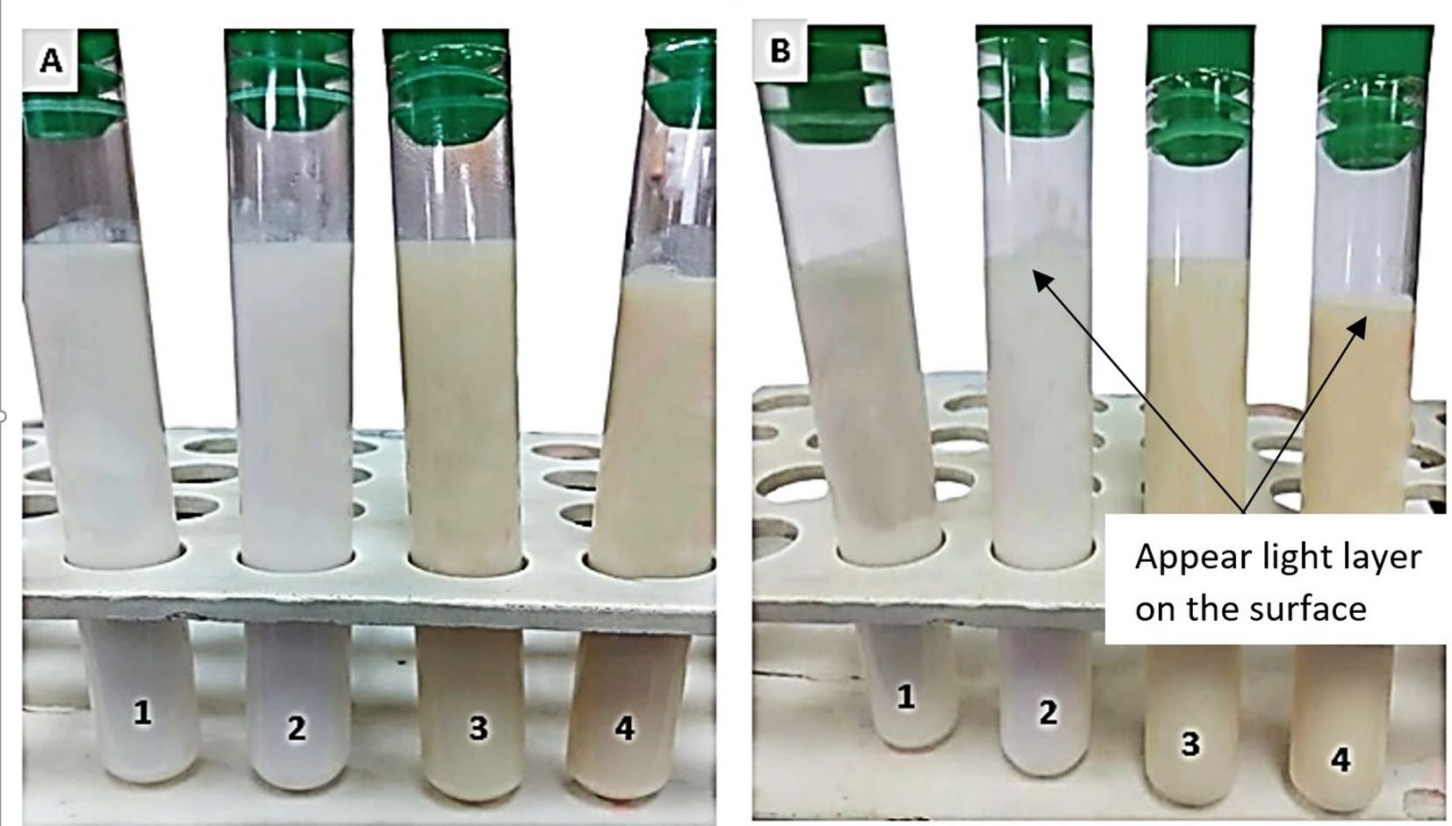
Optical photographs of the MSO/BSO prepared nanoemulsions with concentrations of; 5% MSO (1), 10% MSO (2), 5% BSO (3) and 10% BSO (4). (A) Fresh nanoemulsions; (B) After 7 days of storage at 25 °C. MSO, Moringa seed oil; BSO, Black seed oil.

Optical photographs were captured to visually evaluate the creaming index of freshly prepared (1 A) and 7-day stored (1B) oil nanoemulsions, as depicted in (Fig. 1). The prepared concentrations of MSO and BSO nanoemulsion, precisely 5% MSO, 10% MSO, 5% BSO, and 10% BSO, exhibited no significant differences (*P* < 0.05) regarding their creaming stability indices. The nanoemulsion preparations established with a 10% whey protein isolate (WPI) exhibited enhanced stability. Additionally, no creaming formation was observed for the following 7 days of storage period at room temperature. The creaming stability results strongly correlated with the zeta potential, as the absence of sedimentation or creaming was only observed in nanoemulsions with a zeta potential value exceeding − 35 mV.

Therefore, to succeed in nanoemulsions preparations that exhibit optimal stability in terms of creaming, it is necessary to attain an appropriate concentration close to the saturation surface coverage value. The procedure yields a satisfactory quantity of protein that effectively enhances surface coverage. Additionally, it pertains to stabilizing electrostatic forces, thereby preventing coalescence and creaming^4^.

### Microstructure of prepared nanoemulsions (TEM)

**Fig. 2:**
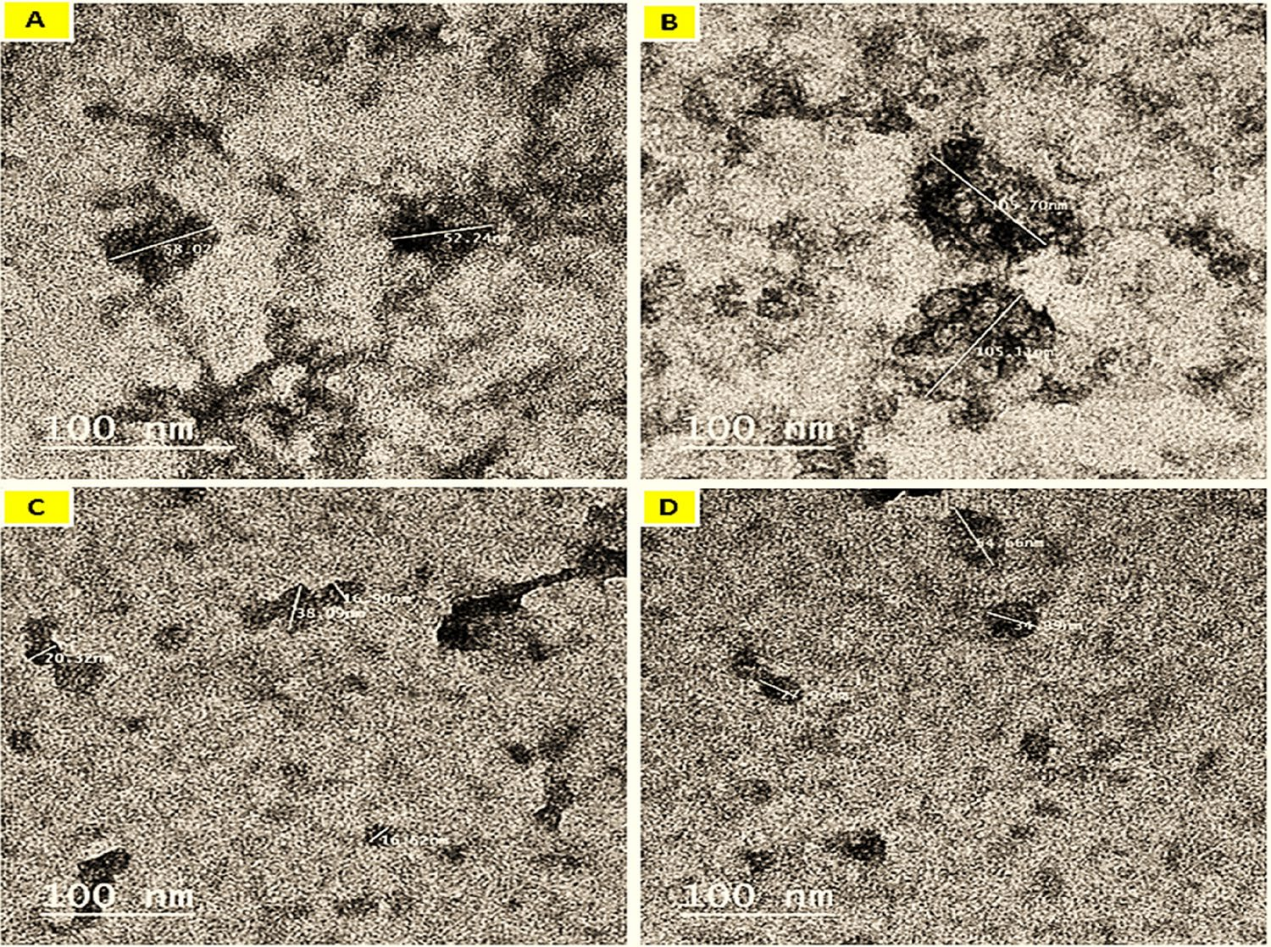
Transmission electron micrographs of nanoemulsions (100 nm, 200,000X, 100 kV). A, 5% MSO; B, 10% MSO; C, 5% BSO; D, 10% BSO.

The morphological characteristics of a 10% whey protein isolate nanoemulsion made with 5% and 10% (w/v) MSO or BSO are shown in Fig. 2. Since the negative-staining procedure was used, the protein fractions that were fixed and covered in a layer of stain were shown by the dark regions in each micrograph^39^. Semi-globular-shaped WPI-BSO or WPI-MSO droplets with diameters ranging from around 69.06 to 135.90 nm were generated, as shown in TEM images. The droplet size rose when the MSO or BSO concentration addition was raised from 5% t 10% (Fig. 2B, D). The TEM image of nanoemulsion containing 10% BO or MSO showed a size increase (Fig. 2A, C). Figures 2B, D illustrates the round, light-colored patches encased in protein that were oil droplets. These droplets were made with 10% ol MSO or BSO and 10% WI, which had a semi-spherical form and a smooth surface produced inside of it.

Additionally, the irregularly shaped oil droplets were assembled around WPI. While in Figure 2A, C, which was synthesized with 5% oil MSO or BSO. There were more black, semi-spherical particles in this nanoemulsion. Because there may be a lot of shell material (WPI). The emulsion contains all of the spherical droplets that are nano-size; most of the small droplets clumped together and stuck to the solution’s protein components. The modified whey protein isolate served as the wall, and the MSO or BSO oil droplets as the core of these droplets, which maintained their spherical capsule structures and hence a core-shell structure. Related research found that higher concentrations of wheat germ oil were integrated into the whey protein via hydrophobic interactions, leading to the formation of spherical whey protein particles^40^. Due to discrepancies between the hydrodynamic size determined by DLS and the dehydrated size seen by TEM, the size estimated by TEM was less than that acquired by Zeta-sizer^41^.

### Cytotoxicity assessment of prepared nanoemulsions

The cytotoxicity assessment (hemolysis%) of prepared nanoemulsions of concentrations 5% and 10% of MSO and BSO are illustrated in Table 3. Hemolysis represents the most commonly employed initial toxicity assessment^42^. The results revealed that the percentage of hemolysis increased by either increasing concentrations of the oil (5% ad 10%) r increasing the same oil concentration (from 5 to 100 µg/mL). The results revealed that the four nanoemulsions preparations are better to be kept less than concentration of (80 µg/mL) to keep the hemolysis percent less than 10%. hen comparing the same concentration of the two applied oils; moringa seed oil showed higher cytotoxicity than the black seed oil with significantly lower IC_50_ (405.45, 857.69 mg/ mL) at concentration of 5% and (337.88, 365.57 mg/ mL) at concentration of 10% rspectively.

The haemolytic activity results were used as a guide for safe fortification based on^15,43^, who stated that haemolysis values below 10% are cnsidered non-haemolytic while values above 25% are considered hemolytic. Consequently, the two forms of applied fortification concentrations in this study were of concentration of 5% of both nanoemulsions and kept less than the recommended range (80 µg/mL) to ensure that % hemolysis are within the safe range.

**Table 3:** Cytotoxicity assessed as hemolytic activity of prepared nanoemulsions.

Concentration (µg/mL)	Hemolysis %			
	5% MSO	10% MSO	5% BSO	10% MSO
5	1.12^c^ ± 0.02	2.91^a^ ± 0.12	0.22^d^ ± 0.03	1.57^b^ ± 0.07
10	3.14^b^ ± 0.12	3.81^a^ ± 0.19	0.45^c^ ± 0.05	4.04^a^ ± 0.24
40	4.04^b^ ± 0.22	4.26^b^ ± 0.15	2.47^c^ ± 0.10	6.50^a^ ± 0.31
80	4.71^c^ ± 0.28	5.38^b^ ± 0.32	4.48^c^ ± 0.33	9.19^a^ ± 0.42
100	12.33^c^ ± 0.38	14.80^a^ ± 0.49	5.83^d^ ± 0.46	13.68^b^ ± 0.52
IC_50_ (µg/ml)	405.45^b^ ± 0.01	337.88^d^ ± 0.03	857.69^a^ ± 0.01	365.57^c^ ± 0.04

Data represented as means of triplicate ± SD.

MSO, Moringa seed oil; BSO, Black seed oil.

Means followed by different letters are significantly different (*P* ≤ 0.05).

### Physicochemical properties of fortified yoghurt

Table 4 shows the results of chemical composition of fresh fortified yoghurt samples. Obtained results showed no significant effects of nanoemulsions fortification on total solids (TS), protein and fat content comparing T1 the control sample and the fortified yoghurt samples (T2, T3, T4 and T5). On the other hand, the nanoemulsions fortification showed significant decrease in T3, T5 samples’ acidity that showed the least acidity (2.00, 2.50% respectively) comparing to T1 (2.50%) and free oil fortified samples T2, T4 (2.50, 2.70% respectively).

Table 5 illustrated physicochemical properties of fortified yoghurt when fresh and after 15 days of cold storage. The results showed that the pH values decreased for all samples to end up with values ranged between (4.46–4.28) at the end of the storage period. This indicates that nanoemulsions did not show negative effect on starter culture lactic acid bacteria viability. T3 fortified with MSO nanoemulsion showed the highest initial pH (4.56), which came in accordance with the titratable acidity value (2%).

Table 5 shows the syneresis and the water holding capacity (WHC) of fortified Yoghurt samples. Syneresis is the drainage of whey from yoghurt curd during storage, conversely, WHC is the ability of yoghurt curd to bind water into its protein network. High values of WHC are concomitant with the low rates of syneresis, which is preferred in yoghurt and reflected on the products acceptability. Therefore, manufacturers tend to add thickening agents and texture modifiers to control syneresis and WHC. The complex chemical composition of nanoemulsion initiates interactions with the fortified Yoghurt constituents. However, the WHC decreased and syneresis tended to be increased after 15 days of storage in all yoghurt samples.

The Color analyses of fortified Yoghurt are illustrated in (Table 6). T3 showed to be the lightest color among the fortifications (*L* = 77.85) which was comparable to control (*L* = 77.74). However, the two nanoemulsion fortifications T3 and T5 (77.85, 77.29) tended to be lighter than their extract oil fortifications T2, T4 (77.25, 76.66) as the latest was the darkest due to the BSO dark color. Masking the colors applying the nano-preparations was previously supported by^44^. The relationship of particle size to color is well known and is mathematically quantified by scattering theories. Such properties are affected by reduced dimensionality of nanoparticles, as the distributions of nanoparticles are used to tune the index of refraction^45^. All fortified yoghurt samples were located in the area between –*a* and *b* that indicates yellowish green. T4 showed to be the darkest greenish color which matched the sensory evaluation as it recorded the least scores (Fixx. 8).

**Table 4:** Chemical composition of fortified yoghurt.

Parameters	T1	T2	T3	T4	T5
TS	15.50 ^c^ ± 0.78	17.07^b^ ± 0.28	18.50^a^ ± 0.42	17.05^b^ ± 0.45	18.43^a^ ± 0.61
Protein	4.45^b^ ± 0.05	4.38^c^ ± 0.04	5.67^a^ ± 0.06	4.39^bc^ ± 0.02	5.65^a^ ± 0.6
Fat	3.20^c^ ± 0.28	4.65^ab^ ± 0.15	4.60^b^ ± 0.05	4.70^a^ ± 0.14	4.65^ab^ ± 0.28
Acidity	2.60^ab^ ± 0.07	2.50^b^ ± 0.22	2.00^d^ ± 0.14	2.70^a^ ± 0.28	2.30^c^ ± 0.00

T1: Control plain yoghurt; T2: yoghurt fortified with free MSO; T3: yoghurt fortified with MSO nanoemulsion; T4, yoghurt fortified with free BSO; T5, yoghurt fortified with BSO nanoemulsion.

TS: total solids.

Data are represented in means (g/100 g) ± SD.

Means with different superscript small letters (a-e) within the same row are statistically different (*p* < 0.05).

**Table 5:** Physicochemical properties of fortified Yoghurt.

Storage (days)	T1	T2	T3	T4	T5
pH					
1	4.57^bA^ ± 0.03	4.51^cA^ ± 0.01	4.65^aA^ ± 0.03	4.51^cA^ ± 0.01	4.62^abA^ ± 0.01
5	4.53^abA^ ± 0.03	4.52^abA^ ± 0.04	4.59^aA^ ± 0.06	4.45^bB^ ± 0.04	4.56^aB^ ± 0.03
10	4.41^bcB^ ± 0.01	4.45^bB^ ± 0.04	4.55^aAB^ ± 0.00	4.32^cC^ ± 0.04	4.51^abC^ ± 0.01
15	4.37^bcB^ ± 0.06	4.39^bB^ ± 0.08	4.46^abB^ ± 0.04	4.28^cC^ ± 0.02	4.49^aC^ ± 0.03
Syneresis%					
1	17.50^bD^ ± 0.25	15.40^cD^ ± 0.14	10.20^dD^ ± 0.28	24.80^aD^ ± 0.04	7.90^eC^ ± 0.20
5	18.90^bC^ ± 0.28	17.30^cC^ ± 0.42	11.70^dC^ ± 0.14	27.40^aC^ ± 0.14	8.60^eB^ ± 0.14
10	20.40^bB^ ± 0.57	19.70^bB^ ± 0.28	13.90^cB^ ± 0.00	28.20^aB^ ± 0.28	9.50^dAB^ ± 0.70
15	21.30^bA^ ± 0.27	20.40^bA^ ± 0.25	14.40^cA^ ± 0.28	32.10^aA^ ± 0.85	10.20^dA^ ± 0.14
WHC%					
1	85.60^dA^ ± 0.85	87.50^cA^ ± 0.71	90.50^bA^ ± 0.42	84.50^dA^ ± 0.71	93.10^aA^ ± 0.14
5	81.80^bB^ ± 1.55	77.80^cAB^ ± 0.71	87.90^aB^ ± 0.14	81.60^bB^ ± 1.41	88.50^aB^ ± 0.71
10	79.40^cBC^ ± 0.85	73.40^dB^ ± 0.57	86.30^bC^ ± 0.28	80.20^cBC^ ± 0.28	87.90^aB^ ± 0.42
15	75.50^dC^ ± 0.71	71.50^eC^ ± 1.41	82.60^bD^ ± 0.85	78.30^cC^ ± 0.28	85.20^aC^ ± 0.28

Data are represented in means ± SD.

Lowercase letters indicate significance between different treatments, while uppercase letters indicate significance between time intervals of the same treatment (*p* > 0.05). WHC, Water holding capacity.

T1, Control plain Yoghurt; T2, Yoghurt fortified with free MSO; T3, Yoghurt fortified with MSO nanoemulsion; T4, Yoghurt fortified with free BSO; T5, Yoghurt fortified with BSO nanoemulsion.

**Table 6:** Color analyses of fortified yoghurtl.

Parameters	T1	T2	T3	T4	T5
L*	77.74^a^ ± 0.04	77.25^c^ ± 0.21	77.85^a^ ± 0.45	76.66^ab^ ± 0.57	77.29^c^ ± 0.14
a*	-1.30^b^ ± 0.30	-1.28^b^ ± 0.13	-1.13^a^ ± 0.14	-1.25^ab^ ± 0.16	-1.14^a^ ± 0.10
b*	5.29^b^ ± 0.49	5.00^c^ ± 0.57	4.74^d^ ± 0.45	6.33^a^ ± 0.17	5.27^b^ ± 0.03
*∆E*	0.00 ± 0.00	0.57^b^ ± 0.01	0.59^b^ ± 0.02	1.50^a^ ± 0.07	0.48^c^ ± 0.05

Data represented as means ± SD.

^a, b,^. Means in the same row followed by different superscript letters differ significantly (*P* > 0.05).

L*, value measuring black (0)/white (100); a*, value measuring green (-)/red (+); b*, value measuring blue (-)/yellow (+).

T1, Control plain Yoghurt; T2, yoghurt fortified with free MSO; T3, yoghurt fortified with MSO nanoemulsion; T4, yoghurt fortified with free BSO; T5, yoghurt fortified with BSO nanoemulsion.

### Fatty acid profile of fortified yoghurt and development during storage

**Fig. 3:**
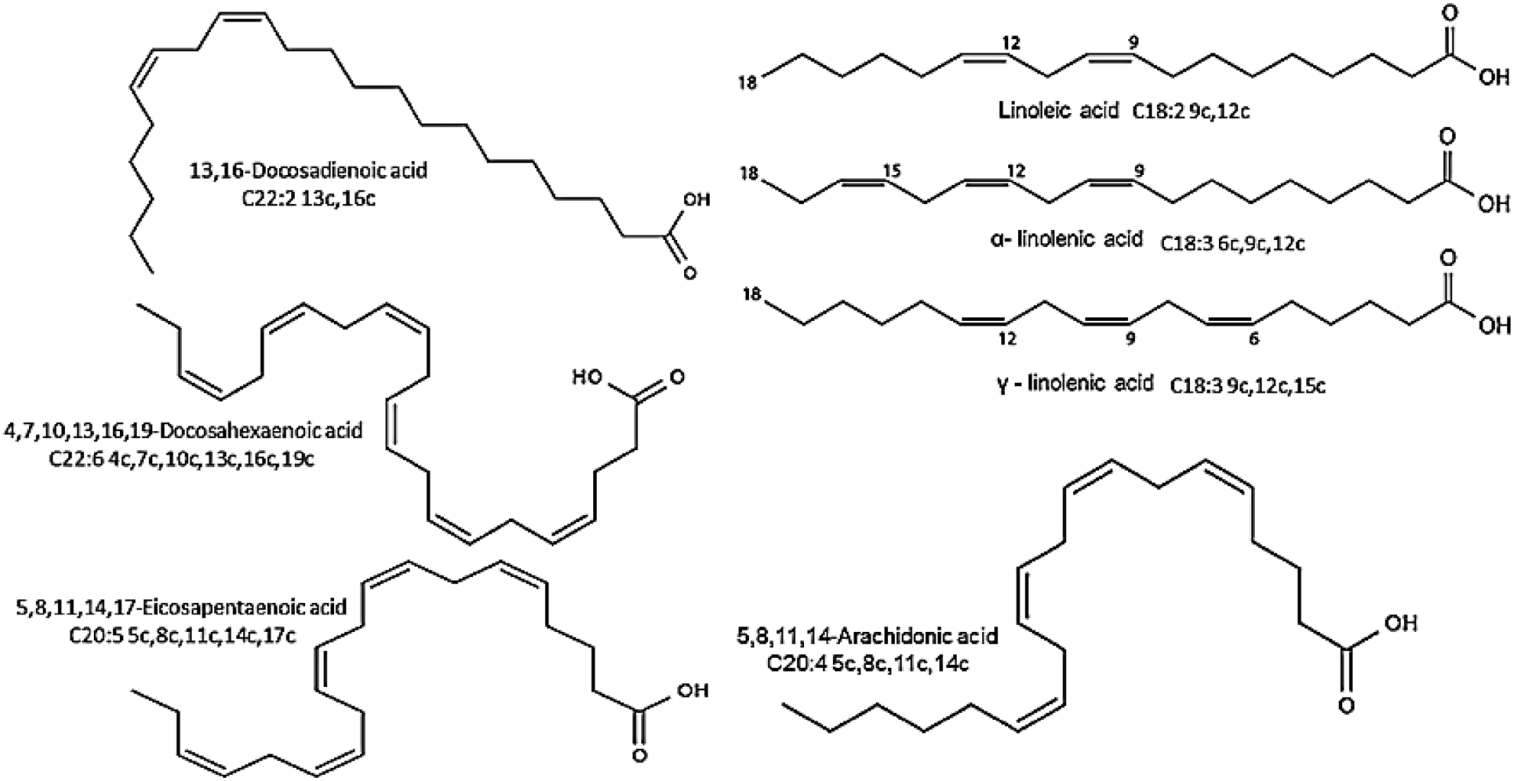
Representative fatty acids tentatively detected in the fortified yoghurt samples using GC–MS spectrometer.

Yoghurt flavor compounds such as esters are produced from fat hydrolysis during storage, especially in the presence of nanoemulsions/ oil extracts. LAB plays a crucial role in fat degradation and the production of aroma compounds. For evaluation of the fatty acid profile, fortified yoghurt samples were analyzed using the GC-MS technique when fresh and after 15 days of cold storage. Results of the fatty acids profile are presented in Tables 7 and 8; Figure 3. In this study, 21 fatty acids were detected in zero time: 12 saturated fatty acids (SFAs), 6 monounsaturated fatty acids (MUFAs) (Omega 9), and 3 polyunsaturated fatty acids (PUFAs) (Omega 6, 3) (Table 7). The major SFAs identified were palmitic acid (38.81, 33.95, 33.16, 34.53, 29.68%), myristic acid (11.24, 9.04, 8.60, 9.09, 7.17%), and stearic acid (9.23, 8.45, 8.25, 8.03, 7.07%) in T1, T2, T3, T4, and T5, respectively. The obtained results indicated that the control yoghurt T1 showed higher concentration of SFAs comparing with the fortified yoghurt, which align with the saturated fatty acids content in milk that accounts 70% by weight, where the dominant fatty acids are palmitic acid (16:0) (30% by weight of the total fatty acids), followed by myristic acid (14:0) and stearic acid (18:0) (11 and 12% by weight, respectively)^46^. On comparing USFAs; the highest MUFAs compound was oleic acid that showed concentrations of (22.00, 26.99, 27.51, 21.03, 24.08%) especially in T3 fortified with MSO nanoemulsion, while linoleic acid was the highest compound of the PUFAs especially in T5 fortified with BSO nanoemulsion with content of (3.07, 7.45, 8.26, 14.91, 21.10%) or T1-T5 respectively. Moringa oil is characterized by a high content of oleic acid (75.39–77.40%)^5^, while the fatty acid profile of black seed oil reported the concentration of linoleic acid to be the highest (58.9%), with prominent oleic (28.1%), palmitic (12.5%), and stearic (3.1%) acids, respectively^44^. Additionally, it is well known that esters are the main cause of fruity flavors. In fortified yoghurt samples, methyl butanoate and methyl octanoate were detected. These results were confirmed by other results reported by Rahim and coauthors^47^.

**Table 7:** Fatty acid profile of fresh fortified yoghurt samples.

Common name	Lipid numbers	Relative area (%)				
		T1	T2	T3	T4	T5
Saturated fatty acids SFAs						
Caproic acid	C6:0	1.26 ± 0.03	0.97 ± 0.01	0.93 ± 0.03	0.95 ± 0.02	0.75 ± 0.02
Caprylic acid	C8:0	1.05 ± 0.02	0.83 ± 0.02	0.79 ± 0.02	0.84 ± 0.01	0.67 ± 0.03
Capric acid	C10:0	2.52 ± 0.05	2.06 ± 0.06	1.94 ± 0.07	2.06 ± 0.05	1.64 ± 0.05
Undecanoic acid	C11:0	0.30 ± 0.01	0.23 ± 0.02	0.23 ± 0.01	0.24 ± 0.01	0.20 ± 0.01
Lauric acid	C12:0	3.61 ± 0.08	2.94 ± 0.09	2.72 ± 0.06	2.91 ± 0.07	2.24 ± 0.04
Tridecanoic acid	C13:0	0.16 ± 0.01	0.13 ± 0.01	0.13 ± 0.01	0.14 ± 0.02	0.11 ± 0.01
Myristic acid	C14:0	11.24 ± 0.22	9.04 ± 0.19	8.60 ± 0.09	9.09 ± 0.10	7.17 ± 0.08
Pentadecanoic acid	C15:0	0.97 ± 0.07	0.78 ± 0.04	0.74 ± 0.05	0.79 ± 0.06	0.61 ± 0.02
Palmitic acid	C16:0	38.81 ± 1.52	33.95 ± 1.01	33.16 ± 1.07	34.53 ± 1.05	29.68 ± 1.11
Heptadecanoic acid	C17:0	0.45 ± 0.02	0.38 ± 0.01	0.36 ± 0.03	0.29 ± 0.01	0.22 ± 0.02
Stearic acid	C18:0	9.23 ± 0.33	8.45 ± 0.25	8.25 ± 0.18	8.03 ± 0.31	7.07 ± 0.17
Arachidic acid	C20:0	0.11 ± 0.02	0.27 ± 0.02	0.54 ± 0.07	0.08 ± 0.01	0.21 ± 0.01
Monounsaturated fatty acids MUFAs, Omega-9						
Myristoleic acid	C14:1 9c	0.85 ± 0.01	0.69 ± 0.02	0.66 ± 0.02	0.69 ± 0.03	0.55 ± 0.01
Palmtoleic acid	C16:1 9c	1.76 ± 0.07	1.48 ± 0.04	1.45 ± 0.03	1.52 ± 0.04	1.26 ± 0.03
cis-10-Heptadecanoic acid	C17:1 10c	0.27 ± 0.02	0.25 ± 0.02	ND	ND	0.11 ± 0.01
trans-9-Elaidic acid	C18:1 9t	1.32 ± 0.03	1.48 ± 0.05	1.57 ± 0.04	0.58 ± 0.02	0.99 ± 0.02
Oleic acid	C18:1 9c	22.00 ± 0.89	26.99 ± 0.89	27.51 ± 1.02	21.03 ± 1.00	24.08 ± 0.98
cis-11-Eicosenoic acid	C20:1 11c	0.14 ± 0.01	0.20 ± 0.02	0.28 ± 0.02	0.13 ± 0.01	0.12 ± 0.01
Polyunsaturated fatty acids PUFAs, Omega 6,3						
Linoleic acid	C18:2 9c,12c	3.07 ± 0.35	7.45 ± 0.69	8.26 ± 0.72	14.91 ± 0.52	21.10 ± 1.03
γ-Linolenic acid	C18:3 6c,9c,12c	0.08 ± 0.01	0.08 ± 0.01	0.23 ± 0.01	0.04 ± 0.01	0.03 ± 0.01
α-Linolenic acid	C18:3 9c,12c,15c	ND	ND	0.89 ± 0.01	0.43 ± 0.01	ND
∑SFAs		69.72 ± 0.75	60.04 ± 0.84	58.39 ± 0.97	59.94 ± 0.91	50.55 ± 0.86
∑UFAs		29.49 ± 0.89	38.62 ± 0.95	40.86 ± 1.08	39.33 ± 0.99	48.23 ± 1.21
∑MUFAs		26.34 ± 0.76	31.10 ± 0.89	31.48 ± 1.00	23.95 ± 0.69	27.11 ± 0.88
∑PUFAs		3.15 ± 0.45	7.53 ± 0.67	9.38 ± 0.81	15.38 ± 0.89	21.13 ± 1.01
PUFAs: MUFAs ratio		11.95 ± 0.78	24.21 ± 0.98	29.79 ± 1.12	64.21 ± 1.10	77.94 ± 1.25
UFAs: SFAs ratio		42.29 ± 1.00	64.34 ± 1.05	69.99 ± 1.87	65.61 ± 1.03	95.41 ± 1.42

SFAs, saturated fatty acids; MUFAs, monounsaturated fatty acids; PUFAs, polyunsaturated fatty acids.

T1, Control plain Yoghurt; T2, Yoghurt fortified with free MSO; T3, Yoghurt fortified with MSO nanoemulsion; T4, Yoghurt fortified with free BSO; T5, Yoghurt fortified with BSO nanoemulsion. Data represented as means ± SD.

After 15 days of storage (Table 8); the fatty acid profile was changed to 33 detected fatty acids; 15 saturated fatty acids (SFAs), 8 monounsaturated fatty acids (MUFAs) (Omega 9) and 10 polyunsaturated fatty acids (PUFAs) (Omega 6, 3). No great differences were observed in fatty acids contents as; palmitic acid content was (37.98, 32.96, 34.65, 32.39, 29.23%), myristic acid (10.86, 8.05, 9.11, 8.69, 7.15%) and stearic acid (9.23, 7.84, 7.86, 7.29, 6.60%). Similarly, in UFAs where oleic acid concentrations were (23.90, 28.52, 26.96, 23.61, 23.78%), and linoleic acid content was (2.83, 9.62, 7.67, 15.397, 22.13%) for T1-T5 respectively. Nevertheless, new MUSFs and PUSFs appeared after storage that were not observed in fresh products such as 2 MUSFs; erucic acid (C22:1 13c) and nervonic acid (C24:1 15c), and 7 PUSFs namely; eicosadienoic acid (C20:2 11c,14c), eicosatrienoic acid (C20:3 8c,11c,14c), eicosatrienoic acid (C20:3 11c,14c,17c), arachidonic acid (C20:4 5c,8c,11c,14c), eicosapentaenoic acid (C20:5 5c,8c,11c,14c,17c), docosadienoic acid (C22:2 13c,16c) and docosahexaenoic acid (C22:6 4c,7c,10c,13c,16c,19c). Significant variations especially in long chain PUFAs composition were previously reported during storage, in addition to some identified differences in contents of linoleic, linolenic, and arachidonic acids that were changed significantly. This confirms that the storage period represents a main factor in the chemical composition changes in seed oils/ seed oils fortifications^3^. These results indicate the relation between the microbial community and the fatty acid profile changes, due to the microbial interaction with yoghurt and the abundance of other unknown influencers that are yet to be examined^48^.

The observed changes were reflected on increased PUFAs: MUFAs ratio that was (11.95, 24.21, 29.79, 64.21, and 77.93) in fresh yoghurt vs. (12.98, 35.34, 28.85, 60.71, and 86.84) after storage, and UFAs: SFAs ratio (42.29, 64.33, 69.98, 65.60, and 95.40) vs. (47.13, 76.57, 65.95, 77.40, and 100.79).

Currently it is not possible with conventional GC–MS methods without the use of additional spectroscopic analyses to detect isomers. Despite developments to produce more accurate information with mass spectra, isomers and especially ring-positional isomers can still yield very similar MS spectra. This brings the need for more powerful methods to perform spectral comparison than traditional MS library searches against known spectra. Multivariate data analysis could be one of the promising chemometric approach to extract and visualize small differences in mass spectra of isomers^49^. Theoretically, we assume that this could explain that the UFAs: SFAs ratio after 15 days of storage exceeded 100% (100.79) which could be relied to overlapping assessment in fatty acids isomers.

**Table 8:** Fatty acid profile of fortified yoghurt samples after 15 days of storage.

Common name	Lipid numbers	Relative area (%)				
		T1	T2	T3	T4	T5
Saturated fatty acids SFAs						
Caproic acid	C6:0	1.11 ± 0.03	0.82 ± 0.01	1.01 ± 0.02	0.93 ± 0.01	0.82 ± 0.02
Caprylic acid	C8:0	0.99 ± 0.01	0.73 ± 0.02	0.86 ± 0.01	0.80 ± 0.02	0.71 ± 0.02
Capric acid	C10:0	2.47 ± 0.2	1.81 ± 0.02	2.08 ± 0.03	1.96 ± 0.05	1.66 ± 0.02
Undecanoic acid	C11:0	0.30 ± 0.01	0.22 ± 0.01	0.25 ± 0.02	0.24 ± 0.02	0.21 ± 0.01
Lauric acid	C12:0	3.49 ± 0.02	2.59 ± 0.01	2.89 ± 0.03	2.79 ± 0.01	2.24 ± 0.02
Tridecanoic acid	C13:0	0.17 ± 0.01	0.12 ± 0.01	0.14 ± 0.01	0.13 ± 0.01	0.12 ± 0.01
Myristic acid	C14:0	10.86 ± 0.41	8.05 ± 0.47	9.11 ± 0.43	8.69 ± 0.54	7.15 ± 0.39
Pentadecanoic acid	C15:0	0.93 ± 0.01	0.69 ± 0.01	0.77 ± 0.02	0.74 ± 0.02	0.60 ± 0.01
Palmitic acid	C16:0	37.98 ± 1.03	32.96 ± 0.57	34.65 ± 0.95	32.39 ± 0.48	29.23 ± 0.45
Heptadecanoic acid	C17:0	0.36 ± 0.01	0.30 ± 0.01	0.31 ± 0.02	0.29 ± 0.01	0.21 ± 0.01
Stearic acid	C18:0	9.23 ± 0.23	7.84 ± 0.21	7.86 ± 0.25	7.29 ± 0.29	6.60 ± 0.19
Arachidic acid	C20:0	0.07 ± 0.00	0.24 ± 0.00	0.15 ± 0.01	0.14 ± 0.01	0.23 ± 0.01
Heneicosanoic acid	C21:0	0.01 ± 0.00	ND	ND	ND	ND
Docosanoic acid	C22:0	ND	0.23 ± 0.00	0.14 ± 0.00	ND	0.03 ± 0.01
Lignoceric acid	C24:0	ND	0.04 ± 0.00	0.01 ± 0.00	ND	0.01 ± 0.00
Monounsaturated fatty acids MUFAs, Omega-9						
Myristoleic acid	C14:1 9c	0.83 ± 0.02	0.62 ± 0.00	0.71 ± 0.01	0.67 ± 0.01	0.56 ± 0.01
Palmtoleic acid	C16:1 9c	1.78 ± 0.01	1.40 ± 0.01	1.59 ± 0.02	1.47 ± 0.01	1.29 ± 0.01
10-Heptadecanoic acid	C17:1 10c	0.17 ± 0.01	0.14 ± 0.01	0.14 ± 0.00	0.14 ± 0.02	0.12 ± 0.01
9-Elaidic acid	C18:1 9t	1.66 ± 0.02	1.12 ± 0.01	1.25 ± 0.02	1.13 ± 0.01	0.97 ± 0.01
Oleic acid	C18:1 9c	23.90 ± 0.22	28.52 ± 0.31	26.96 ± 0.45	23.61 ± 0.15	23.78 ± 0.17
cis-11-eicosenoic acid	C20:1 11c	ND	0.25 ± 0.01	0.16 ± 0.01	0.12 ± 0.01	0.15 ± 0.01
Erucic acid	C22:1 13c	0.02	ND	ND	ND	ND
Nervonic acid	C24:1 15c	0.01 ± 0.00	0.62 ± 0.01	0.01 ± 0.00	ND	0.01 ± 0.00
Polyunsaturated fatty acids PUFAs, Omega-6,3						
Linoleic acid	C18:2 9c,12c	2.83 ±	9.62 ± 0.52	7.67 ± 0.49	15.40 ± 0.98	22.13 ± 0.89
γ-Linolenic Acid	C18:3 6c,9c,12c	0.05 ± 0.01	0.09 ± 0.01	0.09 ± 0.01	0.03 ± 0.01	0.03 ± 0.00
α-Linolenic acid	C18:3 9c,12c,15c	0.46 ± 0.02	1.34 ± 0.01	0.84 ± 0.01	0.43 ± 0.01	0.43 ± 0.00
Eicosadienoic acid	C20:2 11c,14c	0.02 ± 0.00	0.05 ± 0.01	0.03 ± 0.01	0.43 ± 0.01	0.63 ± 0.01
Eicosatrienoic acid	C20:3 8c,11c,14c	0.07 ± 0.01	0.06 ± 0.01	0.08 ± 0.01	0.06 ± 0.00	0.08 ± 0.01
Eicosatrienoic acid	C20:3 11c,14c,17c	0.05 ± 0.01	0.01 ± 0.00	0.01 ± 0.00	ND	0.01 ± 0.00
Arachidonic acid	C20:4 5c,8c,11c,14c	0.19 ± 0.01	0.13 ± 0.01	0.15 ± 0.00	0.13 ± 0.01	ND
Eicosapentaenoic acid	C20:5 5c,8c,11c,14c,17c	0.01 ± 0.00	0.02 ± 0.00	0.03 ± 0.00	0.014 ± 0.01	0.02 ± 0.01
Docosadienoic acid	C22:2 13c,16c	ND	0.01 ± 0.00	ND	ND	ND
Docosahexaenoic acid	C22:6 4c,7c,10c,13c,16c,19c	ND	ND	7.67 ± 0.01	15.40 ± 0.03	0.01 ± 0.00
∑SFAs		67.97 ± 1.25	56.63 ± 1.05	60.23 ± 1.14	56.37 ± 1.12	49.81 ± 0.98
∑UFAs		32.03 ± 0.87	43.37 ± 0.97	39.72 ± 0.85	43.63 ± 0.79	50.20 ± 0.89
∑MUFAs		28.35 ± 0.98	32.04 ± 0.94	30.83 ± 0.88	27.15 ± 0.79	26.87 ± 0.82
∑PUFAs		3.68 ± 0.19	11.32 ± 0.25	8.89 ± 0.17	16.48 ± 0.27	23.33 ± 0.29
PUFAs: MUFAs ratio		12.98 ± 0.31	35.34 ± 0.35	28.85 ± 0.30	60.71 ± 52	86.84 ± 0.98
UFAs: SFAs ratio		47.13 ± 0.40	76.57 ± 1.01	65.95 ± 0.88	77.40 ± 0.99	100.79 ± 1.59

SFAs, saturated fatty acids; MUFAs, monounsaturated fatty acids; PUFAs, polyunsaturated fatty acids.

SFAs, saturated fatty acids; MUFAs, monounsaturated fatty acids; PUFAs, polyunsaturated fatty acids.

“Isomeric fatty acids” is a term that refers to those fatty acids in which the double bonds originally present are modified in either conformation (cis-/trans-) or position by partial hydrogenation of naturally occurring oils^50^. Figure 3 illustrates representative fatty acids tentatively detected in the fortified yoghurt samples using GC–MS spectrometer.

Obtained results reviled that applied fortifications with oil seeds/ oil seeds nanoemulsions enhanced flavor development of fortified Yoghurt, in addition to increased functional properties with their higher content of PUFAs with all well-known health benefits.

### Antioxidant potentials of fortified yoghurt

**Fig. 4:**
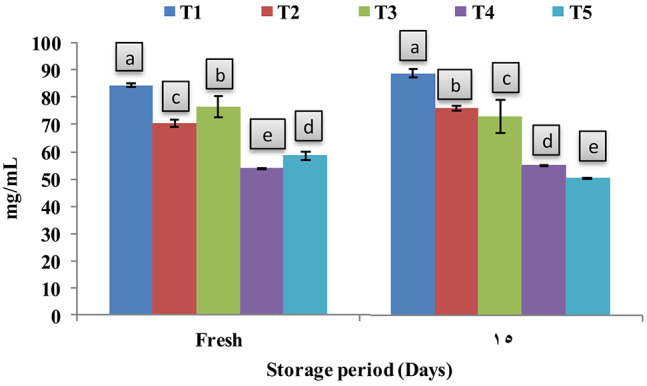
Antioxidant potentials represented as IC_50_ (mg/ mL). Data represented are means of duplicates ± SD. IC_50_, the inhibitory concentration at which 50% of DPPH radical is scavenged. T1, Control plain yoghurt; T2, yoghurt fortified with free MSO; T3, yoghurt fortified with MSO nanoemulsion; T4, yoghurt fortified with free BSO; T5, yoghurt fortified with BSO nanoemulsion.

The estimated antioxidant potentials of the yoghurt products with different fortifications were analyzed when fresh and after 15 days of cold storage. Figure 4 showed the increased antioxidant potentials with different oil fortifications, with less IC_50_ values comparing to control (T1). When fresh, the results showed that the oil extract fortifications (T2, T4) showed higher antioxidant potentials (70.38, 53.89 mg/ mL) than both nanoemulsion fortifications T3, T5 (76.49, 58.55 mg/ mL). While on the 15th day of the storage period, a decline in antioxidant potentials in the products fortified with the oil extract T2, T4 (76.01, 55.09 mg/ mL) was noticed. Similar patterns have been reported by^51^. Noteworthy that the nanoemulsions succeeded in enhancing the antioxidant potentials of the T3, T5 products (72.96, 50.30 mg/ mL) during storage, which was reflected in decreased lipid oxidation processes in these yoghurt products. Overall results showed that the two forms of black seed oil BSO/BSO nanoemulsions (T4, T5) showed higher antioxidant potentials than the MSO fortified products (T2, T3). Black seed oil (*Nigella sativa* L.) is rich in phenols which have strong potent antioxidant properties^52^, and *Moringa oleifera* oil was reported with extremely resistance to autoxidation which can be used as an antioxidant for the long-term stabilization with lower levels of peroxides^54^.

### Lipid oxidation prevention

**Fig. 5:**
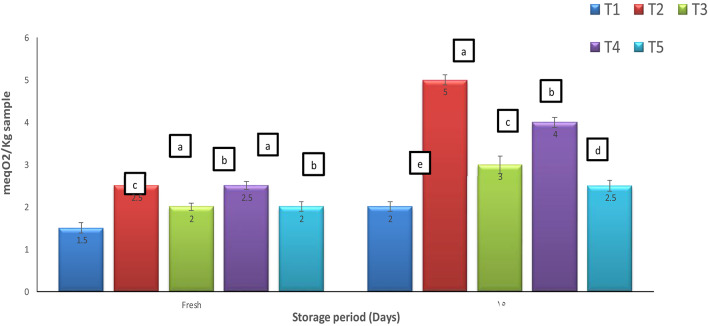
Lipid oxidation represented as peroxide value (meqO_2_/Kg sample). Data represented are means of duplicates ± SD. T1, Control plain Yoghurt; T2, yoghurt fortified with free MSO; T3, yoghurt fortified with MSO nanoemulsion; T4, yoghurt fortified with free BSO; T5, yoghurt fortified with BSO nanoemulsion.

The results of lipid oxidation assessment of the yoghurt products during storage illustrated in (Fig. 5) were correlated to the antioxidant potential (Fig. 4). The black seed oil in both forms showed to be more tolerant to oxidation than MSO due to its higher antioxidant potentials (Fig. 4). The elevation in free radical peroxide oxygen after 15 days of cold storage due to the production of malonaldehyde (MDA), a known marker of the final products of fatty acids peroxidation, indicated that the nanoemulsions made the oils’ antioxidants more capable to protect the products against lipid peroxidation. The products fortified with the nanoemulsions (T3 and T5) showed less lipid oxidation in comparison with the seed oil fortification (T2, T4) due to the effect of nanoemulsions on lipid protection. Lipid oxidation leads to the development of rancidity, off-flavor compounds, and other reactions causing reduction of shelf life, nutritive value and sensory quality. This oxidation process is a major cause of deterioration of edible oil/ edible oil fortified products that make it one of the major concerns in food technology^54^. The protective role of nanoemulsions has been previously documented^44,55^.

### Effect of nanoemulsions on total count of lactic acid bacteria

In the production of yogurt, lactic acid bacteria (LAB), such as *Streptococcus thermophilus* and *Lactobacillus delbrueckii subsp. bulgaricus*, are responsible for the fermentation process (Table 9). The bacteria must stay alive and in significant quantities (at least 10^6^ cfu/g) until the final day of use^56^. The product’s safety is enhanced by acidifying the raw material as organic acids, such as lactic acid, are formed. Like a materials scientist, developing additional metabolites such as ethanol, enzymes, aromatic compounds, bacteriocins, and exopolysaccharides contributes to natural yogurt’s enhanced stability, texture, and nutritional qualities. It is important to ensure that the plant additives are highly quality microbiological. This will help maintain the balance in the starting yogurt and prevent any potential microbial hazards such as bacteria, mold, or fungi^57^.

In the current study, the total LAB count was increased by fortification with essential oil nanoemulsions yogurt (T3 and T5). While the count decreased with Moringa (T2) and Nigella sativa oil (T3) compared with the control. That is due to whey protein isolate (shell of nanodroplet) and MSO or BSO as high-value antioxidants and high PUFA, their reported health benefits and which promoting growth yoghurt starter^58,59^.

When whey proteins are incorporated into yogurt mixes, elevating temperatures during processing enhances the accessibility of amino acids and peptides necessary for the growth of Lactic Acid Bacteria (LAB)^60^, adding peptides and amino acids to be used by LAB and whey protein can impact the pH of growth media^61^.

**Table 9:** Microbiological properties of yoghurt.

Treatment	Storage period (days)	Total bacterial count	LAB	Yeasts & Molds	Coliform	Staphylococci
		log CFU g^− 1^	log CFU g^− 1^			
T1	1	9.66	7.94	ND	ND	ND
	5	9.93	8.84	ND	ND	ND
	10	8.94	8.85	ND	ND	ND
	15	7.74	6.63	1.38	ND	ND
T2	1	8.92	7.71	ND	ND	ND
	5	9.85	8.57	ND	ND	ND
	10	8.76	7.93	ND	ND	ND
	15	7.27	6.78	ND	ND	ND
T3	1	9.89	8.13	ND	ND	ND
	5	9.96	8.95	ND	ND	ND
	10	8.99	8.98	ND	ND	ND
	15	7.82	7.21	ND	ND	ND
T4	1	8.88	7.8	ND	ND	ND
	5	9.86	8.76	ND	ND	ND
	10	8.72	7.91	ND	ND	ND
	15	7.51	6.51	ND	ND	ND
T5	1	9.79	8.24	ND	ND	ND
	5	9.97	8.98	ND	ND	ND
	10	8.93	8.92	ND	ND	ND
	15	8.86	7.15	ND	ND	ND

ND: Not detected.

Data are presented in mean ± SD.

Means with different superscript small letters (a-d) within the same column are statistically different (*p* < 0.05).

Means with different superscript capital letters (A-E) within the same row are statistically different (*p* < 0.05). T1, Control plain Yoghurt; T2, yoghurt fortified with free MSO; T3, yoghurt fortified with MSO nanoemulsion; T4, yoghurt fortified with free BSO; T5, yoghurt fortified with BSO nanoemulsion.

### Microstructural characterization of fortified yoghurt

**Fig. 6:**
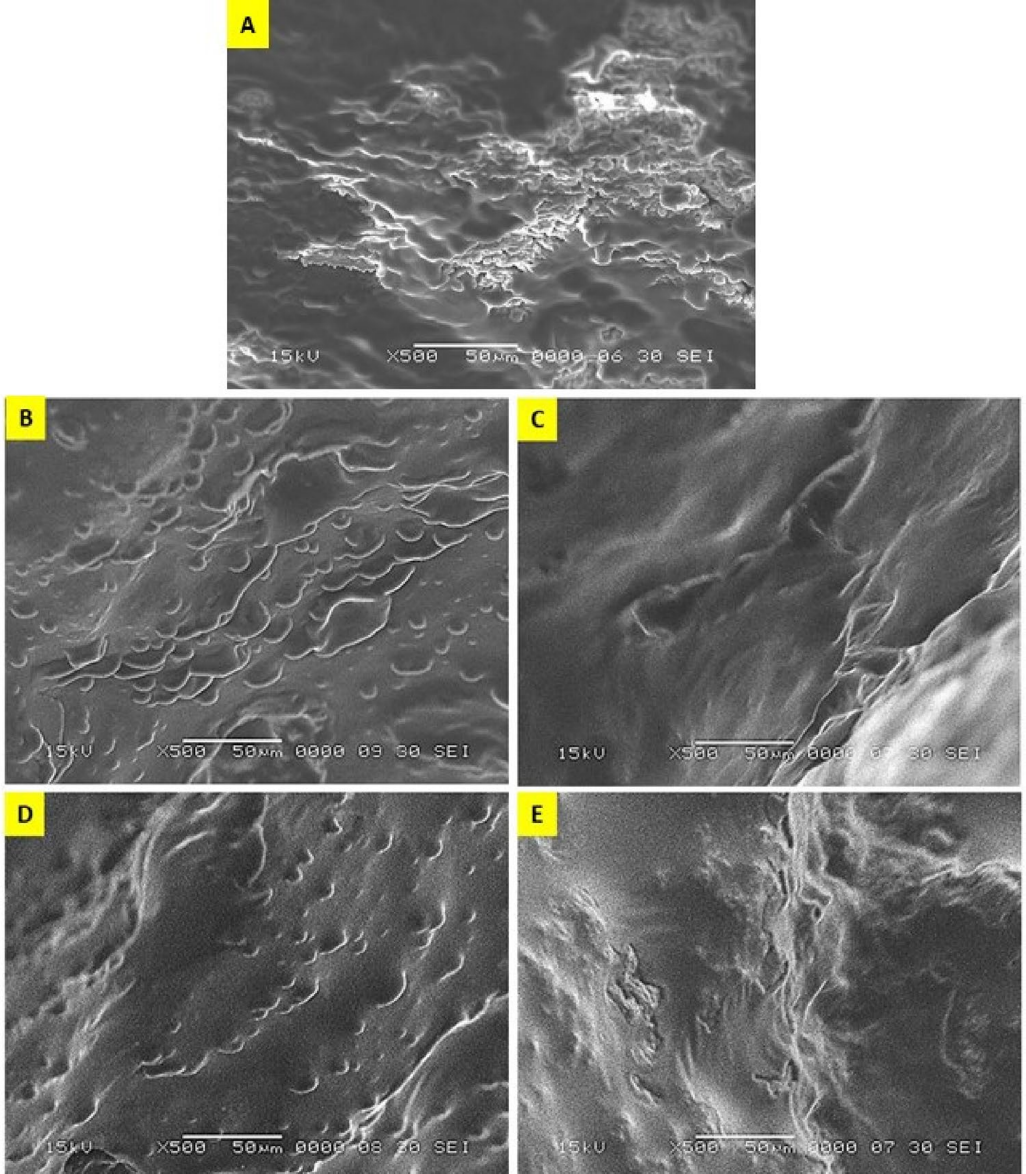
SEM micrographs of fortified yoghurt products (50 μm, X500, 15 kV). A, Control plain Yoghurt(T1); B, Yoghurt fortified with free MSO (T2); C, Yoghurt fortified with MSO nanoemulsion (T3); D, Yoghurt fortified with free BSO (T4); E, Yoghurt fortified with BSO nanoemulsion (T5).

Figures 6 (A, B, C, D and E) illustrate SEM micrographs of fortified yoghurt products; T1, T2, T3, T4 and T5 respectively (50 μm, X500, 15 kV). The oily droplets are observed in Fig. 6B, D^62^, for presence of the MSO/BSO fortifications in T2 and T4 respectively. The MSO/BSO nanoemulsion fortified yoghurt Fig. 6C, E showed soft structure even softer than the control (Fig. 6A) that revealed intact sheets like structure which could significantly affect sensory evaluation as described by El-Kholy and coauthors^44^. These results indicated that nanoemulsions were able to arrange through yoghurt structure to enhance the microstructure and mask the seed oil fortification texture defects. For more images, see supplementary data with the name “samplesidingroups.zip.”

### Texture profile analysis of fortified yoghurt

**Fig. 7:**
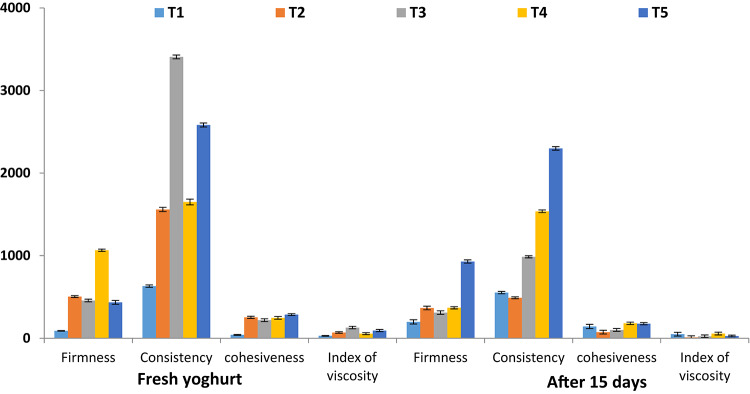
Texture profile analysis of fortified yoghurt. Data are presented in mean ± SD. T1, Control plain Yoghurt; T2, Yoghurtfortified with free MSO; T3, Yoghurtfortified with MSO nanoemulsion; T4, Yoghurtfortified with free BSO; T5, Yoghurtfortified with BSO nanoemulsion.

**Fig. 8:**
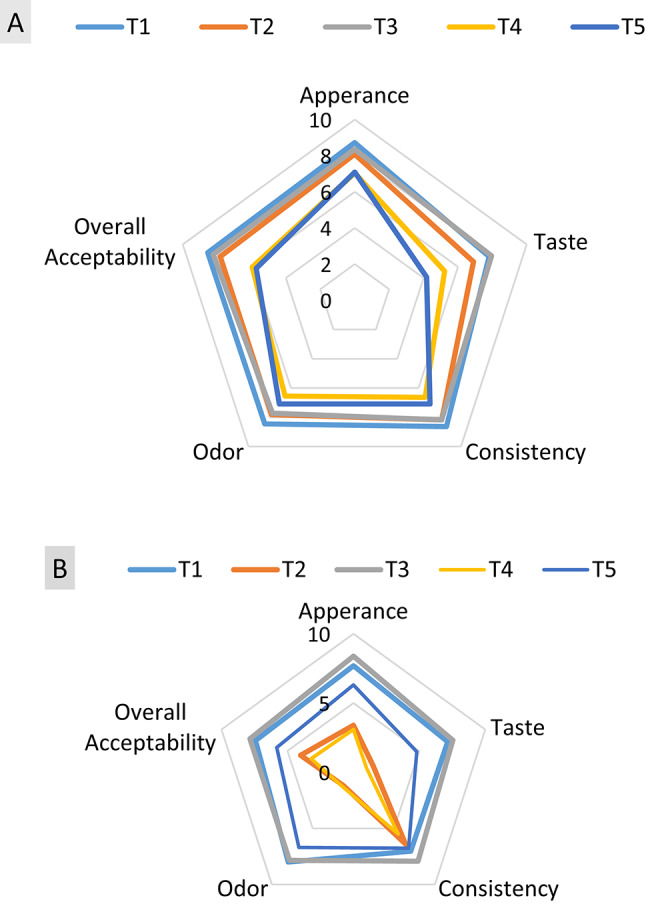
Sensory evaluation of fresh (A) and 15 days stored (B) fortified yoghurt. T1, Control plain Yoghurt; T2, Yoghurt fortified with free MSO; T3, Yoghurt fortified with MSO nanoemulsion; T4, Yoghurt fortified with free BSO; T5, Yoghurt fortified with BSO nanoemulsion.

Texture profile analyses of fortified yoghurt when fresh and after 15 days of cold storage are exhibited in (Fig. 7) representing; firmness (g), consistency, cohesiveness and index of viscosity. When fresh; T4 fortified with BSO showed the highest firmness (1065.99 g), while the 3 other fortified treatments T2, T3 and T5 (504.74 g, 457.28 g, 434.81 g respectively) showed to be higher than plain control (89.94 g). Nanoemulsions showed good impact on yoghurt consistency as, T3 and T5 fortified with MSO, BSO nanoemulsions showed the highest consistency values (3406.94, 2582.79) followed by the oil seeds fortified yoghurt T2, T4 (1559.59, 1650.17) that were higher than plain control T1 (631.16). Same pattern was observed in cohesiveness and index of viscosity where fortified products, T2, T3, T4, T5 showed higher values (253.54, 218.82, 246.79, 285.85) and (69.82, 129.47, 55.86, 93.26 respectively) compared to control (41.12 and 29.47). After 15 days of storage, all texture parameters were decreases, while T5 fortified with BSO nanoemulsion kept good firmness and consistency (929.333, 2298.984). The maximum force of probe is a measure of sample firmness and the area under the peak a measure of consistency. Yoghurt with increased solids (15–16% Table 4) had increased protein content and this causes increase in number of interactions which in turn increases firmness, while cohesiveness indicates structural integrity and is often discussed in terms of the bond strength^63^. Obtained results came in alignment with chemical composition (Table 4), sensory observations (Fig. 8) and in agreement with Salama and coauthors^6^.

### Sensory evaluation of fortified yoghurt

The sensory evaluations of fresh and 15 days stored fortified yoghurt products are illustrated in (Fig. 8). Observed decreases in scores of fresh BSO/ BSO nanoemulsion products; T4 and T5 were recorded in taste, appearance and overall acceptability. This may be due to the high content of phenolic compounds (Table 1) and the dark color of BSO (Table 6). On the contrary, T2 and T3 products fortified with MSO/MSO nanoemulsion scored the best scores of appearance, taste, consistency, odor and overall acceptance that were comparable to control (Fig. 8A). This may be attributed to the less content of MSO in phenolic compounds (Table 1) and its light color (Table 6).

However, after 15 days of cold storage, Fig. 8B highlighted the role of nanoemulsion effect in lipid oxidation protection (Fig. 5), which was pronounced in decreased scores in all sensory parameters of seeds oil fortified yoghurt MSO and BSO, T2 and T4 respectively, due to rancid flavor and color changes. On the other hand, T3 fortified with MSO nanoemulsion maintained the ‘‘highly acceptable” and was comparable to control. Similar observations were reported by Salama and coauthors^6^.

## Conclusion

This study demonstrated that fortification of yoghurt with cold-pressed moringa and black seed oils, especially in nanoemulsion form, significantly enhances the product’s nutritional and functional qualities. The nanoemulsions exhibited excellent physical stability and safety at the tested doses. Fortified yoghurts showed increased unsaturated fatty acid content, improved antioxidant capacity, and maintained viable lactic acid bacteria populations without compromising texture or sensory acceptance. These findings confirm the potential of using moringa and black seed oil nanoemulsions as effective functional ingredients in fermented dairy products.

## Supplementary Information

Below is the link to the electronic supplementary material.

Supplementary Material 1

## Data Availability

All data about this study are presented in the manuscript.
