# Supplementary figures and images for: Enhancing yogurt health benefits with moringa and black seed oil nanoemulsions to improve fatty acids and microbial viability
Source: Sci Rep. 2025 Oct 31;15:38102. doi: 10.1038/s41598-025-22091-2 (PMC12579260; doi:10.1038/s41598-025-22091-2)

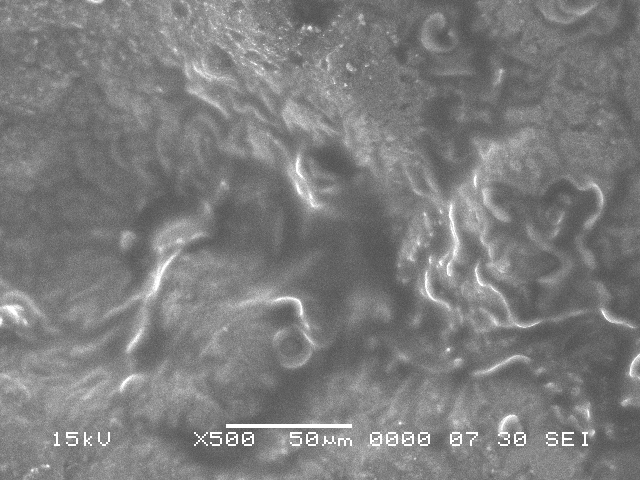

Supplement: Supplementary file 1 — Supplementary Material 1 [file 41598_2025_22091_MOESM1_ESM.zip › C_1-26331-500x.bmp]

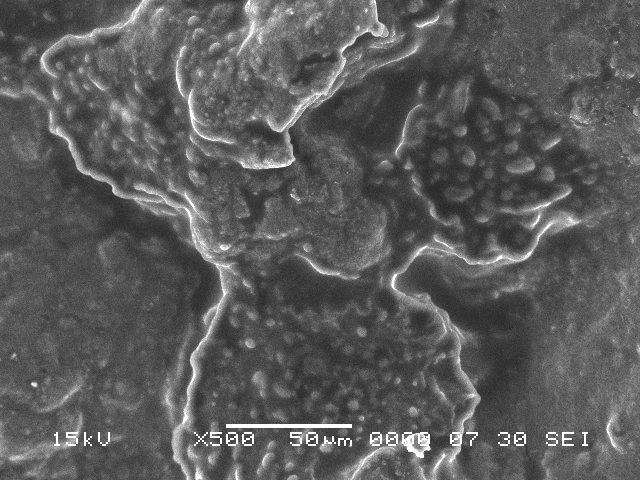

Supplement: Supplementary file 1 — Supplementary Material 1 [file 41598_2025_22091_MOESM1_ESM.zip › C_1-26331-500x1.bmp]

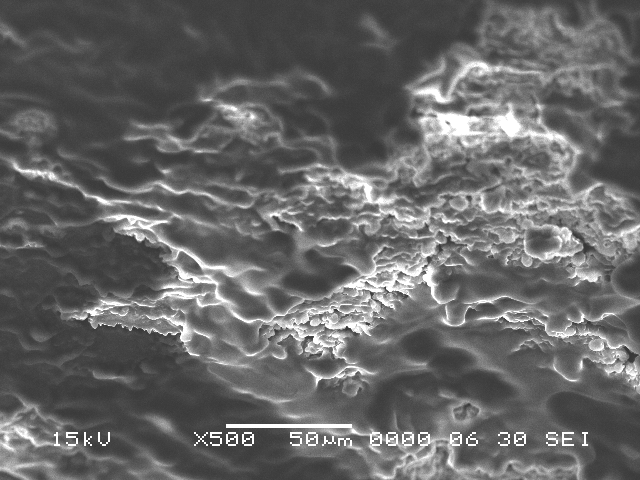

Supplement: Supplementary file 1 — Supplementary Material 1 [file 41598_2025_22091_MOESM1_ESM.zip › C_1-26331-500x2.bmp]

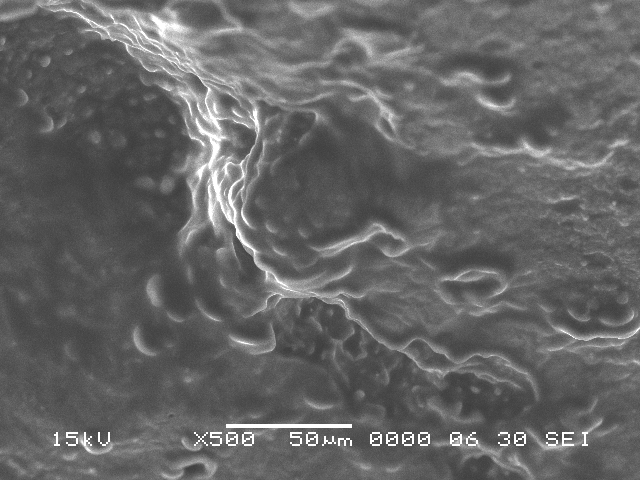

Supplement: Supplementary file 1 — Supplementary Material 1 [file 41598_2025_22091_MOESM1_ESM.zip › C_1-26331-500x3.bmp]

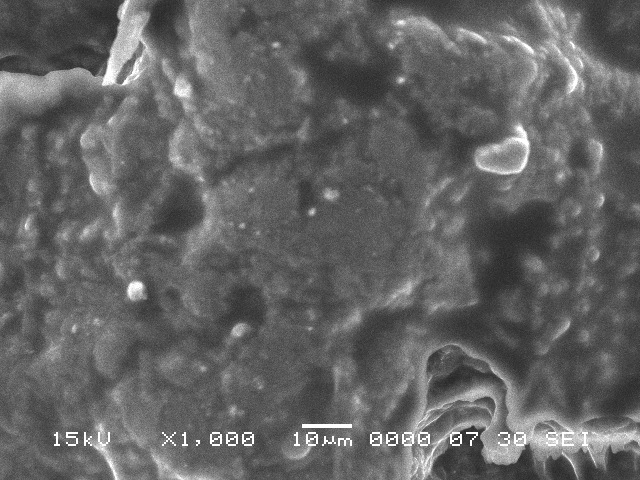

Supplement: Supplementary file 1 — Supplementary Material 1 [file 41598_2025_22091_MOESM1_ESM.zip › C_1-26331-1000x.bmp]

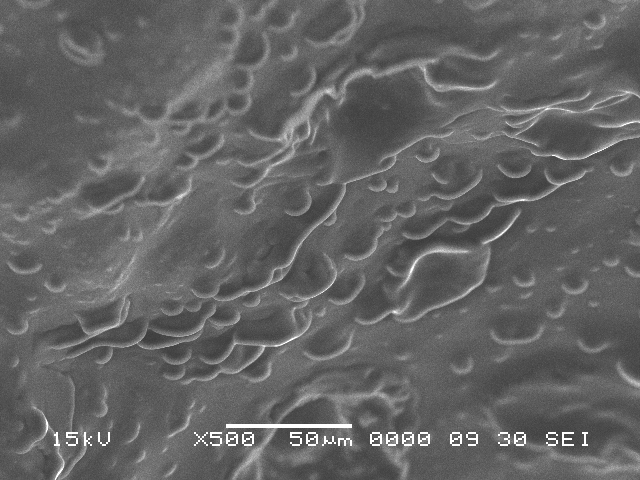

Supplement: Supplementary file 1 — Supplementary Material 1 [file 41598_2025_22091_MOESM1_ESM.zip › M_2-26332-500x.bmp]

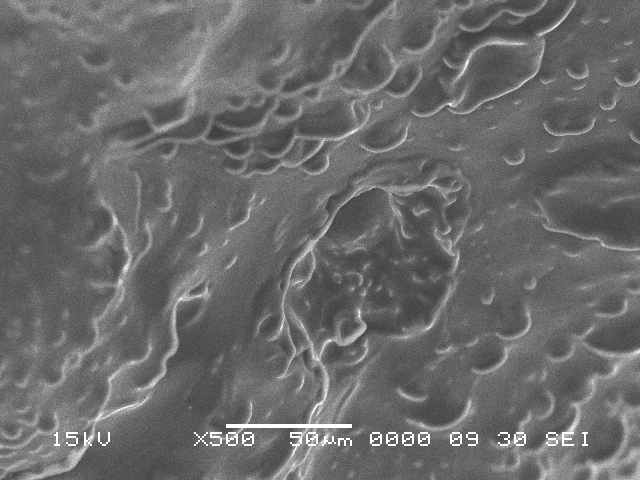

Supplement: Supplementary file 1 — Supplementary Material 1 [file 41598_2025_22091_MOESM1_ESM.zip › M_2-26332-500x1.bmp]

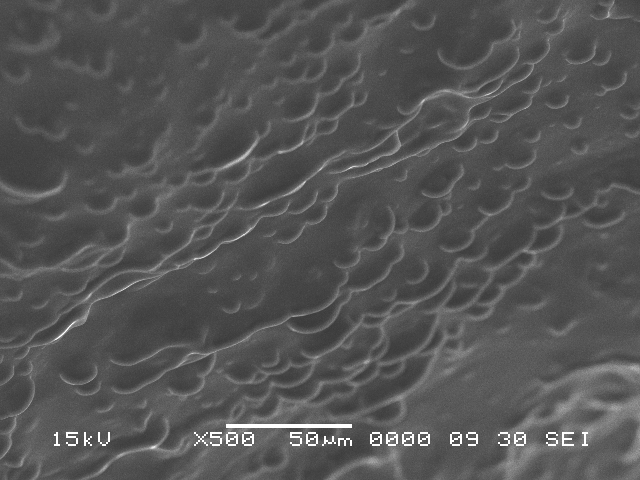

Supplement: Supplementary file 1 — Supplementary Material 1 [file 41598_2025_22091_MOESM1_ESM.zip › M_2-26332-500x3.bmp]

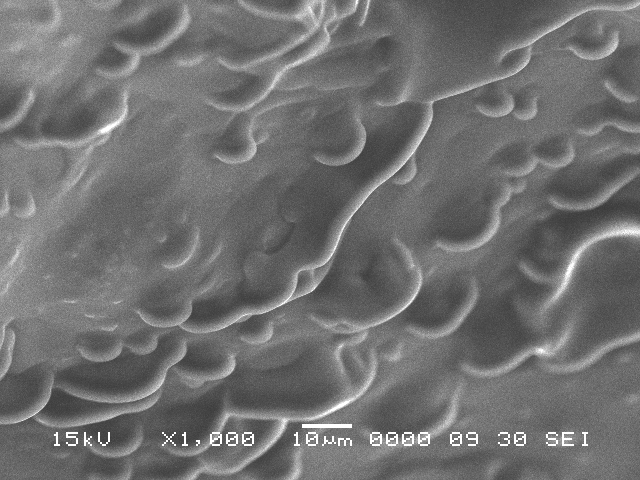

Supplement: Supplementary file 1 — Supplementary Material 1 [file 41598_2025_22091_MOESM1_ESM.zip › M_2-26332-1000x.bmp]

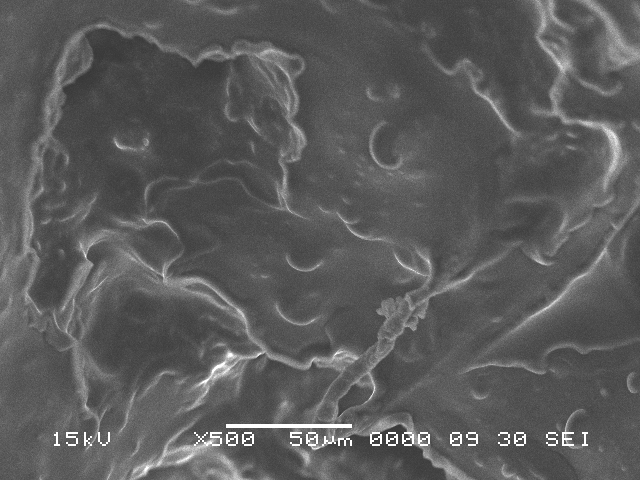

Supplement: Supplementary file 1 — Supplementary Material 1 [file 41598_2025_22091_MOESM1_ESM.zip › M_2-26332-500x2.bmp]

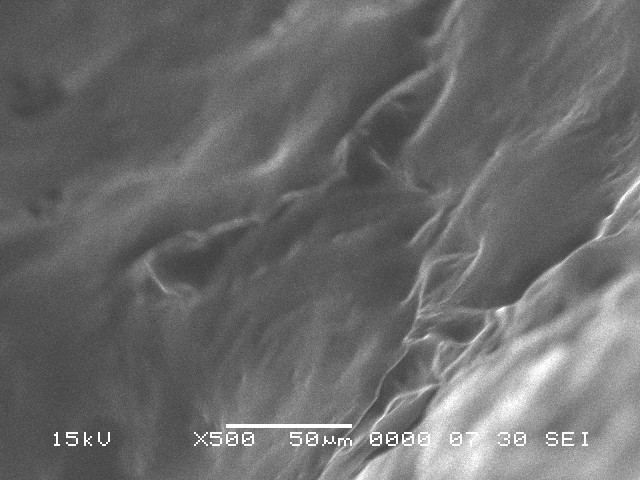

Supplement: Supplementary file 1 — Supplementary Material 1 [file 41598_2025_22091_MOESM1_ESM.zip › Mn_3-26333-500x2.bmp]

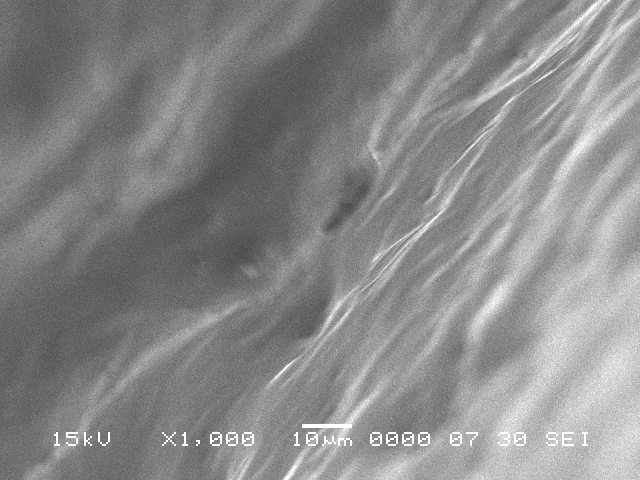

Supplement: Supplementary file 1 — Supplementary Material 1 [file 41598_2025_22091_MOESM1_ESM.zip › Mn_3-26333-500x1.bmp]

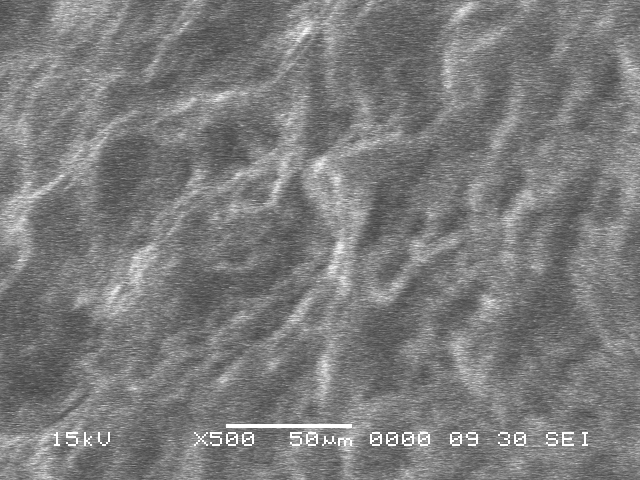

Supplement: Supplementary file 1 — Supplementary Material 1 [file 41598_2025_22091_MOESM1_ESM.zip › Mn_3-26333-500x.bmp]

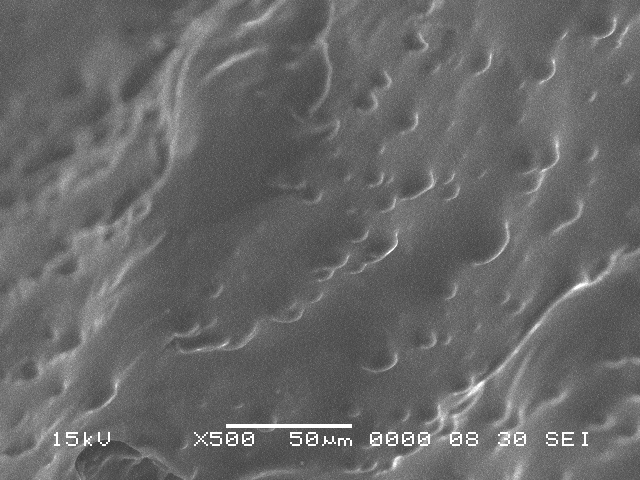

Supplement: Supplementary file 1 — Supplementary Material 1 [file 41598_2025_22091_MOESM1_ESM.zip › B_4-26334-500x.bmp]

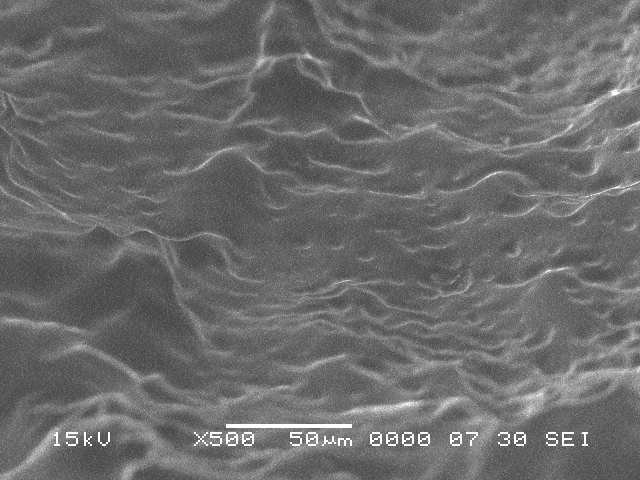

Supplement: Supplementary file 1 — Supplementary Material 1 [file 41598_2025_22091_MOESM1_ESM.zip › B_4-26334-500x1.bmp]

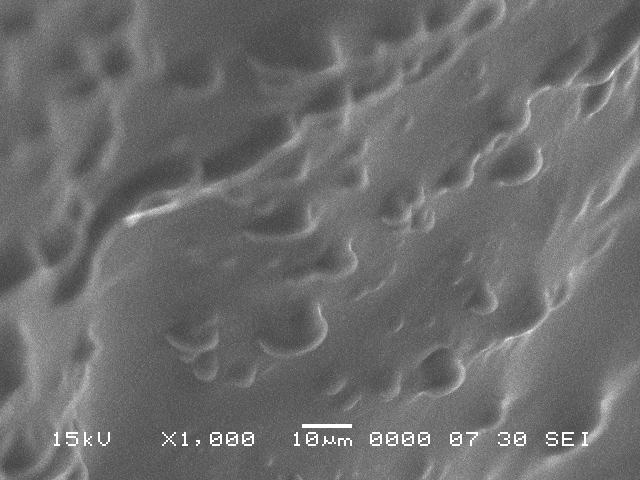

Supplement: Supplementary file 1 — Supplementary Material 1 [file 41598_2025_22091_MOESM1_ESM.zip › B_4-26334-1000x.bmp]

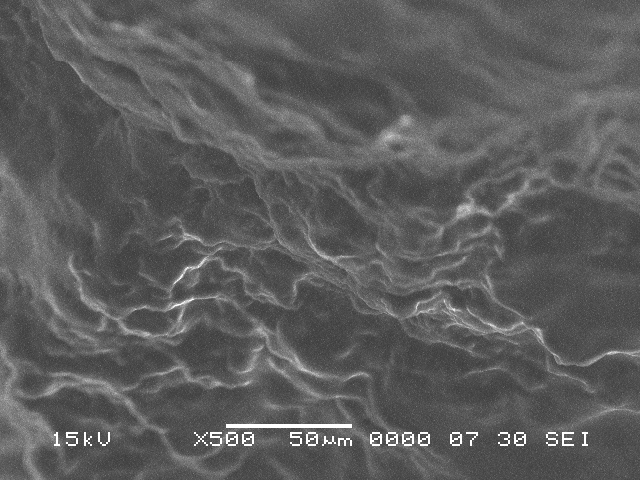

Supplement: Supplementary file 1 — Supplementary Material 1 [file 41598_2025_22091_MOESM1_ESM.zip › B_4-26334-500x3.bmp]

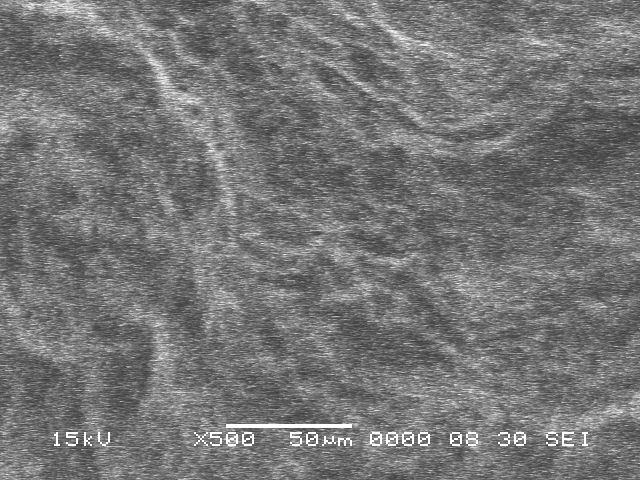

Supplement: Supplementary file 1 — Supplementary Material 1 [file 41598_2025_22091_MOESM1_ESM.zip › Bn_5-26335-500x2.bmp]

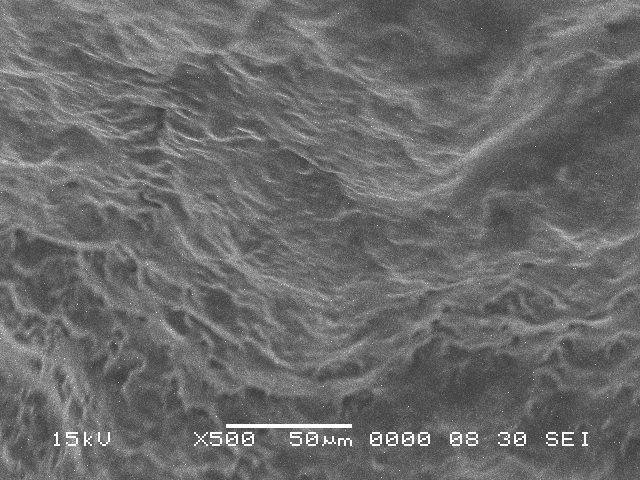

Supplement: Supplementary file 1 — Supplementary Material 1 [file 41598_2025_22091_MOESM1_ESM.zip › Bn_5-26335-500x1.bmp]

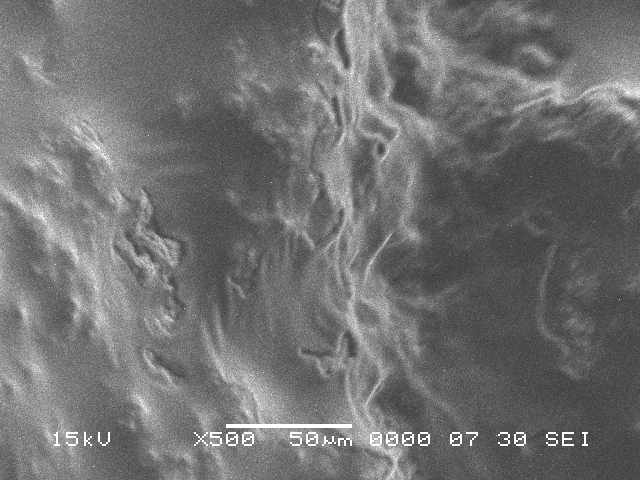

Supplement: Supplementary file 1 — Supplementary Material 1 [file 41598_2025_22091_MOESM1_ESM.zip › Bn_5-26335-500x.bmp]

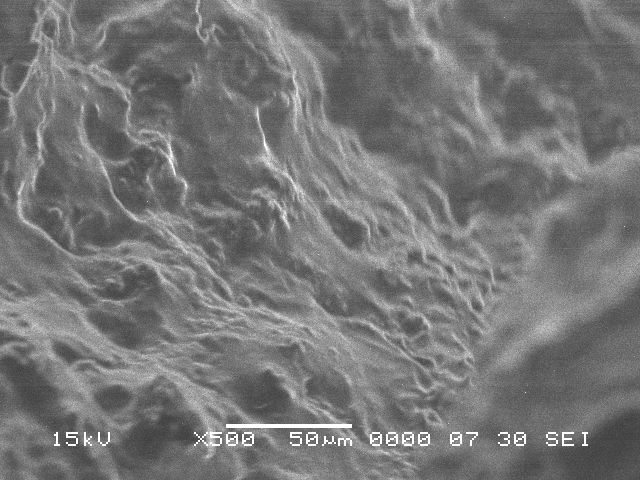

Supplement: Supplementary file 1 — Supplementary Material 1 [file 41598_2025_22091_MOESM1_ESM.zip › Bn_5-26335-500x3.bmp]

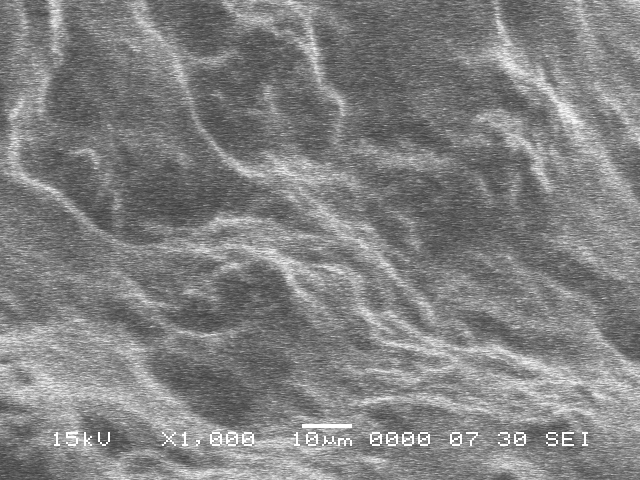

Supplement: Supplementary file 1 — Supplementary Material 1 [file 41598_2025_22091_MOESM1_ESM.zip › Bn_5-26335-1000x.bmp]
